# Work-related injuries among 5 – 17 years-old working children in Egypt: findings from a national child labor survey

**DOI:** 10.1186/s12889-022-13689-6

**Published:** 2022-07-07

**Authors:** Ahmed Mahmoud Fouad, Shaimaa A. A. M. Amer, Yasser Omar Abdellatif, Sally Fawzy Elotla

**Affiliations:** 1 Department of Public Health, Occupational and Environmental Medicine, Faculty of Medicine – Suez Canal University, Kilo 4.5 Ring Road, Ismailia, 41522 Egypt; 2 Center for Global Health Science and Security, Georgetown University, Georgetown, DC USA

**Keywords:** Child labor, Working children, Injuries, Egypt

## Abstract

**Background:**

Egypt has agreed and ratified international regulations that strict child labor. However, the country still struggles with high prevalence of child labor and the associated negative social and health effects. The objective of this study was to identify the prevalence and determinants of work-related injuries among working children in Egypt.

**Methods:**

This study involved a secondary data analysis of the National Child Labor Survey (NCLS) conducted in 2010 by The Central Agency for Public Mobilization and Statistics (CAPMAS) in Egypt with technical and financial support from the ILO’s International Program on the Elimination of Child Labor (IPEC) through its Statistical Information and Monitoring Program on Child Labor (SIMPOC). The total number of working children who responded to questions of work-related injuries in the NCLS child questionnaire was 7485 children.

**Results:**

The prevalence of work-related injuries among working children in Egypt was estimated as 24.1% (95% CI: 22.0%—26.2%), of whom the majority were superficial wounds (87.3%). Among children who reported work-related injuries, 57.9% did not stop work or schooling because of the most serious injury, while 39.6% had stopped temporarily and 2.6% had stopped completely. The main determinants of work-related injuries among working children in the study sample were gender (boys), age of starting work (5–11 years), type of main economic activity (industry and services), type of main workplace (plantation, farms, or garden), the average work hours per week (28 h or more), and exposure to ergonomic and safety, and chemical hazards at work.

**Conclusions:**

The estimated high prevalence of work-related injuries among working children aged 5–17 years in Egypt raises the health risks concerns associated with child labor. Findings of this study on the determinants of work-related injuries could guide policies and interventions to combat child labor and the associated health risks, including work-related injuries.

**Supplementary Information:**

The online version contains supplementary material available at 10.1186/s12889-022-13689-6.

## Background

The International Labor Organization (ILO) had defined child labor as: "work that deprives children of their childhood, their potential and their dignity, and that is harmful to physical and mental development” [1–3]. Children’s work varies from helping families domestically or engagement in a paid economic activity (i.e., children in employment) to a child slavery, where children are taken away from their families and exposed to hazardous conditions or human trafficking [4]. Although many international and national laws that prohibit child labor exist, a tremendous number of children are struggling with child labor. According to the United Nations (UN) in 2020, 218 million children were in employment, of which 152 million were in child labor and 73 million were in hazardous work worldwide [4, 5]. The problem becomes even more apparent in the poorest countries as the number of children between 5–17 years old engaged in child labor surpasses 22 percent [6]. Worldwide, most children work in agriculture, representing 70 percent of all working children between 2012–2016 [7]. By gender, boys and girls are both found to be almost equally affected by child labor, with slightly higher percentages among boys [6].

Child labor has been strongly associated with various health and social consequences. Approximately 50 percent of working children are subject to hazardous work that can significantly affect their development [5]. In the least developed countries, over 25 percent of the children between 5–17 years old are doing work that is perilous to their health [4]. Exposing children to such kinds of works was associated with detrimental health issues, including lack of growth, poor nutrition, increased rates of communicable diseases and illness, behavioral and mental health disorders, and injuries [8–10].

The ILO had estimated that the worldwide incidence of work-related injuries among children as 4.3 percent, or 10 million injured children, with 22,000 deaths every year [11, 12]. In the United States, a child dies and 135 get injured every three days because of agriculture-related work [13]. In Bangladesh, 2.3 percent of working children had work-related injuries [14]. Another study in a brick manufacturing industry in Nepal found working children were eight times more likely to have musculoskeletal issues compared to non-working group [15].

Egypt has agreed and ratified international regulations besides issuing several national laws that strict child labor [16]. However, the country still struggles with a high prevalence of child labor. According to the ILO/ Central Agency for Public Mobilization and Statistics (CAPMAS) National Child Labor Survey (NCLS in 2010, 1.8 million children were in child labor, of whom 1.6 million were in hazardous work [17]. Approximately 63 percent of working children were in the agriculture, while 19 percent were engaged in construction and manufacturing-related work [16]. Earlier studies showed child labor in Egypt was associated with substantial negative social and health consequences. A study in 2019 found a positive correlation between physical and psychological abuse and higher rates of child labor in Egypt [18]. Another study conducted in Assiut, Egypt, showed that financial hardship was a major driver of child labor. The researchers also found that working children of less than 14 years old were more likely to suffer from psychosocial development issues such as anger management and lack of ego strength [19]. Likewise, a study of working children in Alexandria showed that 18.4 percent of working children have had at least one work-related injury, while 7.4 percent had over one injury [20].

There is an ongoing need for evidence on the burden of child labor, associated adverse consequences, and the impact of policies and interventions. This study aimed to estimate the prevalence of work-related injuries and the associated determinants among working children in Egypt; a large fast-growing lower middle-income population that combines the agriculture and industrial communities. We hypothesized that this study could contribute significantly to the existing evidence on the health risks of child labor, guide policies and interventions, and support national and global efforts for combating child labor under the Global Sustainable Strategy and Egypt Vision 2030 [16].

## Methods

This study made use of Egypt’s National Child Labor Survey (NCLS) conducted in 2010 by CAPMAS and ILO International Program on the Elimination of Child Labor (IPEC) through its Statistical Information and Monitoring Program on Child Labor (SIMPOC) [21]. Dataset access and acquisition for secondary analysis was requested from the CAPMAS. In the 2010 NCLS, a national representative sample of about 30,000 households containing children aged 5–17 years was selected from a master sample developed by CAPMAS in early 2010. About 163,628 individuals successfully completed the survey of whom 66,922 children were 5–17 years old (representing about 17.1 million Egyptian children aged 5–17 years). Sampling methods, weights, and survey estimates were explained in NCLS 20l0 report [21].

The questionnaire of the NCLS comprised three main modules: the adult questionnaire, the household characteristics questionnaire, and the child questionnaire (See Supplementary File 1). The adult questionnaire was completed by the most knowledgeable individual in the household to get information about demographic and employment characteristics of all household members above 5 years old. The household characteristics questionnaire was mainly used to assess the household socioeconomic characteristics (e.g., housing characteristics, access to services, and ownership). The child questionnaire was administered to children 5–17 years old and comprised questions about their schooling, engagement in employment, unpaid household services (i.e., chores), and the health and safety conditions of their work.

The study sample was restricted to children in employment or working children aged 5–17 years who reported (NCLS child questionnaire) or reported by their parents (NCLS adult questionnaire), at least one hour of paid or unpaid economic activity in the reference week. Economic activity was defined as any activity that produces a good or services for the purpose of market exchange, own consumption, own-account construction, or production of fixed assets for own use (See Supplementary Files 1 and 2). The total number of working children aged 5–17 year, as reported by either child or parent in the NCLS, was 7772 children, representing 1.8 million Egyptian children [21].

The outcome of interest in this study was work-related injuries among working children. A work-related injury was defined as an injury experienced by a working child because of his/her work in the past 12 months. Working children were asked to self-report on injuries that occurred in the past 12 months because of their work (See Supplementary File 2). Accordingly, the final study sample comprised 7485 working children who had responded to the work-related injuries questions in the NCLS child questionnaire (See Supplementary File 1).

Based on the international conventions and national legislation in Egypt, children in child labor include all children in employment under age 12 years, children 12–14 years who were employed for 14 or more hours per week, and children under 18 years who were engaged in hazardous work. Hazardous exposures in the NCLS questionnaires were categorized into ergonomic and safety hazards, chemical hazards, and physical hazards (See Supplementary File 2).

Data manipulation and statistical analysis were performed with SPSS for Windows, version 25.0 (IBM Corporation, NY, USA). Primary strata (strata), secondary strata (group), and primary sampling units (PSU) variables were used to create a complex samples plan to account for the complex sampling design in the NCLS. SPSS complex samples procedure was used to perform all descriptive, bivariate, and multivariate analyses using the complex samples plan.

Data are presented in tables as unweighted sample frequency, weighted percentages and its standard error, and Odds Ratio (OR) and its 95% confidence interval (CI). We used a logistic regression procedure to test for bivariate and multivariate associations between work-related injuries and study explanatory variables. In bivariate associations, we addressed missing data (i.e., not reported) as a separate category, while in multivariate analysis we excluded all missing data in different variables, except for the “Highest school level” variable, in order not to lose much of the sample (*N* = 6366). Wald Test of Model Effect (F) was used to decide on the statistical significance of each the effect of each explanatory variable. Statistical significance level was set at 5%.

We followed the purposeful model-building strategy [22] to identify the best fit model for understanding the determinants of work-related injuries among the Egyptian working children aged 15–17 years. Testing for collinearity was first performed and showed no collinearity between any of the explanatory variables. The full model included all significant explanatory variables at 0.10 significance level besides the important variables, supported by the literature. The decision of removal of an explanatory variable from the model was based on the significance of explanatory variables at 0.10 significance level, and the significance of difference in -2log L between the smaller and larger models at 0.05 significance level. The final model was the most parsimonious model that showed an insignificant difference in -2log L from the full mode. We also tested for several interactions based on authors’ conceptualization of the study variables and literature, however, they were not statistically significant at 0.10 significance level and consequently we did not keep them in the final model.

## Results

In Table 1, 7485 working children aged 5–17 years old comprised the study sample, of whom children aged 15–17 represented 47.5%. Approximately 76% of the study sample were boys and 84.8% were living in rural areas. The lowest two quintiles of the wealth index (poor and poorest) accounted for 65.5% of all working children. More than half of children were of 5–11 years old when they started work for the first time. Approximately 56% of children combined work, chores, and schooling, while 11.5% were only working. More than half of the children were engaged in unpaid family work and 64.1% were engaged in agriculture-related economic activities. Twenty-one percent of working children were experiencing long working hours (over 42 h per week), while more than half of the children were engaged in hazardous work (ergonomic and safety, chemical and physical hazards). Only 1.8% had received vocational training outside the schools.

**Table 1:** Weighted distribution of work-related injuries in the past 12 months by the sociodemographic and work characteristics among surveyed children (*N* = 7485)

**Characteristics**	**Total surveyed children**		**Work-related injuries** Weighted Row % (SE)	
	Unweighted Frequency	Weighted Column % (SE)	**Yes**	**No**
**Total sample**	7485	100.0 (0.0)	24.1 (1.1)	75.9 (1.1)
**Child age (years)**				
5 – 11	1513	21.3 (0.7)	22.0 (1.7)	78.0 (1.7)
12 – 14	2334	31.1 (0.7)	24.8 (1.5)	75.2 (1.5)
15 – 17	3638	47.5 (0.9)	24.5 (1.2)	75.5 (1.2)
**Child gender**				
Boys	5721	76.4 (0.8)	26.5 (1.2)	73.5 (1.2)
Girls	1764	23.6 (0.8)	16.2 (1.4)	83.8 (1.4)
**Highest completed education level**				
None	650	6.7 (0.5)	25.4 (2.5)	74.6 (2.5)
Primary	1480	15.8 (0.6)	28.4 (1.7)	71.6 (1.7)
Preparatory	637	7.3 (0.5)	24.5 (2.7)	75.5 (2.7)
Secondary or above	118	1.6 (0.2)	28.3 (6.1)	71.7 (6.1)
Not reported	4600	68.5 (1.0)	22.8 (1.3)	77.2 (1.3)
**Place of residence**				
Rural	5489	84.8 (0.8)	23.9 (1.2)	76.1 (1.2)
Urban	1996	15.2 (0.8)	25.1 (1.8)	74.9 (1.8)
**Wealth index quintile**				
Highest (Richest)	239	3.2 (0.4)	12.5 (3.0)	87.5 (3.0)
Fourth	767	10.4 (0.7)	25.0 (2.7)	75.0 (2.7)
Middle	1493	20.9 (1.0)	25.0 (2.1)	75.0 (2.1)
Second	2151	30.6 (1.1)	23.1 (1.8)	76.9 (1.8)
Lowest (Poorest)	2835	34.9 (1.1)	25.2 (1.4)	74.8 (1.4)
**Age at first time to work (years)**				
5 – 11	4055	54.0 (1.1)	25.0 (1.4)	75.0 (1.4)
12 – 14	2134	28.0 (0.9)	23.8 (1.3)	76.2 (1.3)
15 – 17	722	9.0 (0.5)	19.9 (2.2)	80.1 (2.2)
Not reported	574	8.9 (0.7)	23.8 (2.6)	76.2 (2.6)
**Child activities combinations**				
Work only	1046	11.5 (0.6)	27.3 (2.0)	72.7 (2.0)
Work & Chores	1790	19.3 (0.7)	26.0 (1.7)	74.0 (1.7)
Work & Schooling	887	13.3 (0.8)	27.5 (3.0)	72.5 (3.0)
Work, Chores, and Schooling	3756	55.8 (1.1)	21.9 (1.3)	78.1 (1.3)
Not reported	6	0.1 (0.0)	48.9 (23.8)	51.1 (23.8)
**Status in employment**				
Employee	2760	33.2 (1.0)	29.3 (1.4)	70.7 (1.4)
Unpaid family worker	3976	56.8 (1.0)	21.7 (1.3)	78.3 (1.3)
Own account worker or employer	130	1.4 (0.2)	13.3 (3.4)	86.7 (3.4)
Not reported	619	8.7 (0.5)	21.7 (2.2)	78.3 (2.2)
**Main economic activity**				
Agriculture	4606	64.1 (1.4)	23.7 (1.3)	76.3 (1.3)
Industry	2318	28.8 (1.2)	26.8 (1.7)	73.2 (1.7)
Services	550	6.9 (0.5)	16.0 (2.7)	84.0 (2.7)
Not reported	11	0.2 (0.1)	30.1 (13.4)	69.9 (13.4)
**Main workplace**^a^				
Dwelling	870	11.7 (0.7)	15.3 (2.5)	84.7 (2.5)
Plantation, farms, or garden	3519	49.7 (1.2)	26.2 (1.5)	73.8 (1.5)
Service or Sales places	951	11.9 (0.7)	14.4 (1.7)	85.6 (1.7)
Factory or workshops	772	8.7 (0.6)	28.5 (2.4)	71.5 (2.4)
Construction, mines, or quarries	363	4.7 (0.4)	44.8 (4.1)	55.2 (4.1)
Others	393	4.6 (0.4)	23.5 (3.5)	76.5 (3.5)
Not reported	617	8.7 (0.5)	21.7 (2.2)	78.3 (2.2)
**Average work hours (per week)**				
Less than 28 h	4263	60.9 (1.1)	20.7 (1.2)	79.3 (1.2)
28—42 h	1426	18.0 (0.7)	27.9 (2.0)	72.1 (2.0)
Over 42 h	1796	21.0 (0.8)	30.5 (1.9)	69.5 (1.9)
**Exposure to ergonomic and safety hazards**				
No	3078	43.5 (1.2)	9.6 (0.8)	90.4 (0.8)
Yes	4407	56.5 (1.2)	35.2 (1.6)	64.8 (1.6)
**Exposure to chemical hazards**				
No	3677	50.3 (1.3)	12.8 (1.0)	87.2 (1.0)
Yes	3808	49.7 (1.3)	35.5 (1.5)	64.5 (1.5)
**Exposure to physical hazards**				
No	5594	75.6 (1.0)	21.1 (1.2)	78.9 (1.2)
Yes	1891	24.4 (1.0)	33.2 (1.8)	66.8 (1.8)
**Receipt of vocational training**				
No	6738	89.6 (0.5)	24.0 (1.1)	76.0 (1.1)
Yes	133	1.8 (0.3)	38.4 (5.8)	61.6 (5.8)
Not reported	614	8.6 (0.5)	21.4 (2.3)	78.6 (2.2)
**Type of most serious injury (*N* = 1806)**				
Superficial wound	1586	87.3 (1.3)	-	-
Fracture	56	2.6 (0.4)		
Dislocation/Sprain	50	3.4 (0.6)		
Burn	47	2.4 (0.5)		
Eye injury	64	4.2 (0.9)		
**Effect of the most serious injury**^**b**^** (*N* = 1806)**				
No disability	1018	57.9 (2.3)	-	-
Temporary disability	733	39.6 (2.3)		
Permanent disability	55	2.6 (0.5)		

^a^Dwelling: includes family dwelling, others’ dwelling, or client’s places. Service & commercial: includes formal office, shop, kiosk, coffee house, restaurant, hotel, fixed market stall, or in street work. Others: includes different places (mobile), work in pond, lake, river, or other places

^b^Did not stop work or schooling (No disability), Stopped work or school for a short time (temporary disability), Stopped work or school completely (permanent disability)

Table 1 also shows that work-related injuries—in the past 12 months—were reported by 24.1% (95% CI: 22.0%–26.2%), of whom 87.3% were superficial wounds. Approximately 58% of the most serious injuries did not make children stop work or schooling, while 39.6% temporarily stopped work or school and only 2.6% ended up with complete absence from work and school. Work-related injuries were more frequent among older children (> 12 years), boys, children with primary and secondary or older education, children living in urban areas, lower wealth index, starting work before the age of 12 years, children who only work, engaged in employment or unpaid family work, in factories, workshops or farms, longer work hours per week, exposure to hazardous, and with those who had received vocational training (Table 1).

In Table 2, bivariate analyses showed that experiencing work-related injuries was significantly associated with gender (*p* < 0.001), wealth index (*p* = 0.042), child activities combinations (*p* = 0.037), status in employment (*p* < 0.001), type of main economic activity (*p* = 0.022) and workplace (*p* < 0.001), average work hours per week (*p* < 0.001), hazardous exposures (*p* < 0.001) and receipt of vocational training (*p* = 0.013). However, the final most parsimonious model indicated that the only significant determinants of work-related injuries among the studied sample were the gender (*p* = 0.018), age at first time to work (*p* = 0.004), main economic activity (*p* = 0.026), workplace (*p* < 0.001), average work hours per week (*p* = 0.001), exposure to ergonomic and safety hazards (*p* < 0.001), and exposure to chemical hazards at work (*p* < 0.001).

**Table 2:** Bivariate and multivariate analyses of the determinants of experiencing work-related injuries in the past 12 months among surveyed children

	**Bivariate** (*N* = 7485)		**Multivariate (***N* = 6366)			
			**Full Model**^b^		**Final Model**^c^	
	**OR (95% CI)**	***p*-value**	**Adjusted OR (95% CI)**	***p*-value**	**Adjusted OR (95% CI)**	***p*-value**
**Child age (years)**						
5 – 11	1.00 (Reference)	0.230	1.00 (Reference)	0.633		
12 – 14	1.17 (0.97 – 1.41)		1.03 (0.79 – 1.34)			
15 – 17	1.15 (0.95 – 1.39)		0.94 (0.71 – 1.25)			
**Child gender**						
Girls	1.00 (Reference)	< 0.001*	1.00 (Reference)	0.034*	1.00 (Reference)	0.018*
Boys	1.86 (1.54 – 2.25)		1.34 (1.02 – 1.75)		1.35 (1.05 – 1.74)	
**Highest school level—completed**						
None	1.00 (Reference)	0.083	1.00 (Reference)	0.876		
Primary	1.16 (0.87 – 1.56)		1.01 (0.69 – 1.47)			
Preparatory	1.00 (0.66 – 1.39)		0.96 (0.60 – 1.52)			
Secondary or above	1.16 (0.61 – 2.21)		1.36 (0.61 – 3.01)			
Not reported	0.87 (0.65 – 1.16)		0.67 (0.18 – 2.51)			
**Place of residence**						
Rural	1.00 (Reference)	0.569	1.00 (Reference)	0.054		
Urban	1.07 (0.85 – 1.35)		1.31 (1.00 – 1.72)			
**Wealth index quintile**						
Highest (Richest)	1.00 (Reference)	0.042*	1.00 (Reference)	0.717		
Fourth	2.33 (1.27 – 4.26)		1.64 (0.79 – 3.44)			
Middle	2.33 (1.30 – 4.17)		1.37 (0.69 – 2.76)			
Second	2.10 (1.18 – 3.74)		1.38 (0.68 – 2.80)			
Lowest (Poorest)	2.35 (1.34 – 4.09)		1.42 (0.71 – 2.86)			
**Age at first time to work (years)**						
15 – 17	1.00 (Reference)	0.281	1.00 (Reference)	0.013*	1.00 (Reference)	0.004*
12 – 14	1.25 (0.95 – 1.66)		1.28 (0.94 – 1.75)		1.27 (0.94 – 1.71)	
5 – 11	1.34 (0.99 – 1.80)		1.62 (1.16 – 2.27)		1.61 (1.18 – 2.21)	
Not reported	1.26 (0.84 – 1.87)		-		-	
**Child activities combinations**						
Work only	1.00 (Reference)	0.037*	1.00 (Reference)	0.328		
Work & Chores	0.93 (0.73 – 1.20)		1.10 (0.81 – 1.49)			
Work & Schooling	1.01 (0.73 – 1.40)		2.01 (0.53 – 7.62)			
Work, Chores, and Schooling	0.75 (0.58 – 0.95)		1.61 (0.41 – 6.28)			
Not reported	2.55 (0.39 – 16.4)		-			
**Status in employment**						
Own account worker or employer	1.00 (Reference)	< 0.001*	1.00 (Reference)	0.127		
Unpaid family worker	1.81 (1.00 – 3.27)		1.73 (0.85 – 3.52)			
Employee	2.71 (1.50 – 4.87)		1.93 (0.97 – 3.84)			
Not reported	1.81 (0.97 – 3.38)		-			
**Main economic activity**						
Agriculture	1.00 (Reference)	0.022*	1.00 (Reference)	0.053	1.00 (Reference)	0.026*
Industry	1.18 (0.96 – 1.45)		2.27 (1.17 – 4.42)		2.45 (1.28 – 4.70)	
Services	0.61 (0.41 – 0.92)		2.03 (0.95 – 4.33)		2.27 (1.08 – 4.80)	
Not reported	1.39 (0.40 – 4.86)		-		-	
**Main workplace**^a^						
Dwelling	1.00 (Reference)	< 0.001*	1.00 (Reference)	0.001*	1.00 (Reference)	< 0.001*
Plantation, farms**,** or garden	1.96 (1.35 – 2.86)		1.89 (1.22 – 2.93)		1.93 (1.24 – 3.01)	
Service or Sales places	0.93 (0.59 – 1.47)		0.53 (0.28 – 0.99)		0.56 (0.30 – 1.04)	
Factory or workshops	2.20 (1.41 – 3.44)		0.72 (0.39 – 1.31)		0.82 (0.44 – 1.52)	
Construction, mines, or quarries	4.49 (2.82 – 7.15)		1.31 (0.68 – 2.55)		1.41 (0.72 – 2.76)	
Others	1.70 (1.00 – 2.27)		0.78 (0.41 – 1.46)		0.77 (0.40 – 1.51)	
Not reported	1.53 (1.04 – 2.27)		-		-	
**Average work hours (per week)**						
Less than 28 h	1.00 (Reference)	< 0.001*	1.00 (Reference)	0.002*	1.00 (Reference)	0.001*
28—42 h	1.48 (1.21 – 1.81)		1.41 (1.10 – 1.81)		1.38 (1.09 – 1.74)	
Over 42 h	1.68 (1.38 – 2.04)		1.60 (1.21 – 2.12)		1.57 (1.23 – 2.00)	
**Hazardous work exposures**						
Ergonomic and safety hazards	5.08 (4.26 – 6.27)	< 0.001*	3.63 (2.81 – 4.69)	< 0.001*	3.65 (2.84 – 4.68)	< 0.001*
Chemical hazards	3.75 (3.05 – 4.60)	< 0.001*	2.47 (1.94 – 3.14)	< 0.001*	2.46 (1.95 – 3.10)	< 0.001*
Physical hazards	1.85 (1.50 – 2.28)	< 0.001*	0.96 (0.75 – 1.23)	0.738	-	-
**Receipt of vocational training**						
No	1.00 (Reference)	0.013*	1.00 (Reference)	0.106		
Yes	1.97 (1.21 – 3.21)		1.66 (0.90 – 3.08)			
Not reported	0.86 (0.67 – 1.10)		-			

^a^Dwelling: includes family dwelling, others’ dwelling, or client’s places. Service & commercial: includes formal office, shop, kiosk, coffee house, restaurant, hotel, fixed market stall, or in street work. Others: includes different places (mobile), work in pond, lake, river, or other places

^b^Pseudo R Square (Nagelkerke = 0.218), Percent correct classification = 77.1%

^c^Pseudo R Square (Nagelkerke = 0.211), Percent correct classification = 76.3%

^*^Statistically significant *p*-value of the Wald Test of Model Effect (F) at 0.05 level

The odds of work-related injuries were significantly 1.34 times greater for boys relative to girls (95% CI: 1.05 – 1.74). The odds of work-related injuries were significantly 1.61 times greater for children aged 5–11 years compared to children aged 15–17 years (95% CI: 1.18 – 2.21). The odds of work-related injuries were significantly 2.45 and 2.27 times greater for children engaged in industry- and services-related work, respectively, compared to children engaged in agriculture (95% CI: 1.28 – 4.70, and 108 – 4.80, respectively). Likewise, the odds of work-related injuries were significantly 1.93 times greater for work at plantation, farms or garden compared to work at dwelling (95% CI: 1.24 – 3.01). Regarding the hazardous exposures, the odds of work-related injuries were significantly 1.38 and 1.57 times greater for 28–42 work hours and over 42 h per week, respectively, compared to less than 28 work hours per week (95% CI: 1.09 – 1.74, and 1.23 – 2.0, respectively). Likewise, the odds of work-related injuries were significantly 3.65 and 2.46 times greater for children exposed to ergonomic and safety, and chemical hazards, respectively, compared to non-exposed children (95% CI: 2.84 – 4.68, and 1.95 – 3.10, respectively).

## Discussion

This study has estimated the prevalence of work-related injuries among working children in Egypt as 24.1% which was largely higher than the ILO global estimate of 4.3%. The study has also identified children’s gender, age at first time to work, type of economic activity, workplace, average work hours per week, exposure to ergonomic, safety and chemical hazards at work, as the main determinants of work-related injuries among working children in Egypt. These findings contribute significantly to our understanding of the burden of child labor and associated health risks, which support the national and global efforts for combating child labor.

Although the Egyptian child labor law in 1996 bans the employment of children who are less than 14 years of age. The result of the current study revealed that more than half of the working children (54.0%) had started their work early in their life (at 5–11 years), compared by a study in Al-Minia where 73.2% started work below the age of 10 years [23]. In contrast, the monthly labor review by Bureau of Labor Statistics [24] showed that by the ages of 14 and 15 years, the percentage of children working at some types of job increased to 57 and 64 percent, respectively. The Bureau of Labor Statistics [24, 25] reported that hours at work for 16- and 17-year-olds have declined, from a weekly average of 19.7 h in 2000 to 18.0 h in 2004, which compared by higher average in the current study where 60.9% indicated 27 working hours or less and 21.0% 43 h per week, which reflects the low socioeconomic status of child’s family in an attempt to improve their income. Most of the working children in the present study were boys (76.4%) which was consistent with other numerous studies in Jordan [26] Bangladesh (75%) [27], and Al-Minia in Egypt (73%) [23].

In the current study, superficial or open wounds were the most common injuries and comprised 87.3% of all injuries, followed by: eye-related injuries (4.2%), dislocations, strains, or sprains (3.4%), fractures (2.6%), and burns (2.4%). A study conducted in USA (1993) had showed that sprains/strains were the most reported problem (31%), followed by cuts/lacerations (17%), contusions/abrasions (13%), heat burns (8%), and fractures/dislocations (5%) [28]. Another study among children working in the streets of four major cities in Latin America, found that approximately 39.6% of the children reported an injury sustained while working in the streets: scratches (19.5%), cuts/lacerations (16.4%), burns (8.6%), car accidents (8.9%), sprains (4.6%), and amputations (0.3%) [29]. The difference between the current study results and others might be attributed to the nature of work, as most working children in Egypt were engaged in agriculture-related work.

In agriculture work, child laborers under extreme climatic conditions like searing sun and physical hazards for long hours with carrying of heavy loads need more muscular efforts. Lack of enough nutrition with long-lasting awkward body positions, with exposure to chemicals like pesticides and accidents due to using sharps like knives and sometimes heavy dangerous tools and machinery. Besides exposure to insects and wild animals [30–32]. This similarly corresponds with claims of Larson-Bright and his colleges, who observed increased risks of injury for agricultural working children compared to non-working children [33]. The largest proportions of fatalities among youth engaged in agricultural work are associated with the use of farm equipment or occurred because of drowning [34]. High prevalence of child working in agriculture than other occupations as showed by reports of the Human Rights’ Watch, 2006, was estimated that, in developing countries, at least 90% of economically active children in rural areas are employed in agriculture [35].

Other studies in different areas in Egypt concerned with labor among students aged 13–18 years found that 52.7% of students worked in agricultural duties [23, 32, 36]. This percentage is lower when compared with that found by ILO statistics from 20 developing countries. The proportion of children aged 5 to 14 was74% (73.3% of boys and 78.8% of girls) [37]. This could be attributed to in many rural areas where farm work is the main job; many parents believe children will receive more useful training by working on farms than they would in the classroom. As well as in Egypt, economic and social factors have been cited as being responsible for the increasing prevalence of child labor.

Current study results disclosed that the poorest children accounted for most of working population (34.9%) which are consistent with a study in Jordan among working children reported that most children work to learn a skill and help their families (43%, 36% respectively). This makes scarceness and socioeconomic situations as the main reasons for child labor among this study population. This also corresponds with claims of ILO that socioeconomic status has a significant role in exacerbating the child labor phenomenon in the world [37].

A systematic review conducted upon 25 publications about child labor concluded that the physical and mental wellbeing of working children was adversely affected besides other behavioral disorders [8]. Studies investigated the prevalence of general symptoms in working children in Pakistan, Egypt, Lebanon, Jordan, and Indonesia had showed that child labor is negatively associated with health [8]. Watery eyes, chronic cough and diarrhea were common findings, besides the history of a major injury (permanent loss of an organ, hearing loss, bone fractures, permanent disability) that lead to work or school absenteeism [23, 26, 38–40]. Child labor was positively associated with body injury and burns and other health problems [27].

Our results showed that the younger the age for starting work, the greater odds for work-related injuries. Young children and adolescents’ bodies and internal systems are still growing and maturating till age of 18. Although Anatomy, physiology, and psychology differ from adults, these differences may translate into unique risk factors for occupational injuries and illnesses. There is a great variation between adults and children in size. A lack of fit between machines and the physical dimensions and strength of children and adolescents contributes to increase risk of injury. The sleep requirements of children and young youth were different for those in adults, and sleep deprivation can attribute to injuries or occupation illnesses.

Multivariate analysis in this study identified children’s gender, age at initial work, type of economic activity, type of main workplace, long work hours, and exposure to hazardous exposures at workplace (e.g., Ergonomic and safety, and chemical hazards) as the main determinants of work-related hazards among working children. However, a study conducted in Brazil showed that age, gender, smoking, school attendance, sports activities, use of computer/video games/television, domestic activities, care of other children, and care of sick/elderly family members, work activities and workloads associated with musculoskeletal pains and symptoms [41]. Other studies concluded that the lack of work experience, work-related training and supervision increase the risk for work injuries and illnesses among children and adolescents [42, 43].

This study had some limitations that need to be acknowledged. First, identification of work-relatedness of injuries relied only on children’s judgment and ability to recall over a long period (12 months), particularly most of self-reported injuries in this survey were minor injuries, which increases the potential for recall bias. Second, this survey had no data on mechanisms or causes of injuries, which limits the ability to make inference on the risk for occupational injury. Third, a difference between reported economic activities (work) in the past week and work-related injuries in the past 12 months may pose some limitations with the observed association between current work exposures and past (work) injuries. Finally, this national survey is over 10 years old and may pose some limitation for the generalizability of survey findings to the current population. However, this survey is the latest national child labor survey conducted in Egypt, which provides a nationally representative data for about 17.1 million Egyptian children aged 5–17 years.

## Conclusions

Children in employment or working children is a challenging phenomenon in Egypt and other low- and middle-income countries. The global and national struggles in combating child labor emphasize mainly on its worst forms. However, work-related health risks, such as injuries, are important for all forms of work among children. This study highlights the prevalence and determinants of work-related injuries among children of 5–17 years who were engaged in employment. The study estimated the prevalence of work-related injuries as high compared to the global estimates. The study has identified the gender, age at initial work, type of economic activity, type of main workplace, long work hours, and exposure to hazardous exposures at workplace (e.g., Ergonomic and safety, and chemical hazards) as the main determinants of work-related injuries among working children. These findings could guide policies and interventions to combat child labor and the associated health risks, including work-related injuries in Egypt and in other low- and middle-income countries. The findings from this study could inform policies and employment standards for all forms of child work particularly in terms of new regulations or enforcement of existing regulations related to age-specific works, age allowed to work, work hours, the type of work, safety standards and raising the awareness among  individuals, institutions, and society.

## Supplementary Information

**Additional file 1.** National Child Labour Survey 2010 Questionnaire.

**Additional file 2: Table S1.** Framework for identification of child labor (as described in the NCLS official report). **Table S2.** NCLS questionnaire items which were used to identify the work-related injuries. **Table S3.** NCLS questionnaire items which were used to identify working children. **Table S4.** Questions in NCLS questionnaire which describe the workplace hazards.

## Data Availability

The data that support the findings of this study are available from the Central Agency for Public Mobilization and Statistics (CAPMAS) in Egypt, but restrictions apply to the availability of these data, which were used under license for the current study, and so are not publicly available. Data are however available from the authors upon reasonable request and with permission of CAPMAS.
