# Supplementary material for: Work-related injuries among 5 – 17 years-old working children in Egypt: findings from a national child labor survey
Source: BMC Public Health. 2022 Jul 7;22:1303. doi: 10.1186/s12889-022-13689-6 (PMC9260979; doi:10.1186/s12889-022-13689-6)
Supplement: Supplementary file 1 — Additional file 1. National Child Labour Survey 2010 Questionnaire. [file 12889_2022_13689_MOESM1_ESM.pdf]

CAPMAS **Ministry of Manpower & Migration** **ILO**

PSU No. ☐☐☐☐ No. of HH. In PSU ☐☐ HH No. in the Sample Frame ☐☐

NATIONAL CHILD LABOUR SURVEY 2010

GENERAL INFORMATION

Governorate ☐☐

Kism/Markaz ☐☐

/city/ villageShiakha ☐☐☐☐

1- Urban 2- Rural ☐

HH. number on governorate level ☐☐☐☐

HH No. on the level of Shiakha / village ☐☐

Street name/ Bolok No.. :

Building Number... :

Name of HH. Head ....**HONE No.** ☐ **MOBILE NUMBER** ☐

Item	Males	Females	Total
Number of persons in household			
Number of children (5-17)			

If Additional Questionnaires used indicate additional number ☐

This research data is confidential and used only for research purposes

CAPMAS | Ministry of Manpower & Migration | ILO

PSU No. | No. of HH. In PSU | HH No. in the Sample Frame

NATIONAL CHILD LABOUR SURVEY2010

GENERAL INFORMATION

Governorate

Kism/Markaz

/city/ villageShiakha

1- Urban 2- Rural

HH. number on governorate level

HH No. on the level of shiaka/village

Street name/ Bolok No.. :

Building Number.. :

Name of HH. Head ....HONE No. MOBILE NUMBER

INTERVIEWER VISITS					(*) RESULT CODES
**Number of visit**	**Date of visit**	**Time of visit**		**RESULT CODES**	1-Completed<br>2-Completed partially (mention reason)<br>3- No household members at home or no competent respondent<br>4- Entire Household absent for extended period of time<br>5- Dwelling destroyed<br>6- address not a dwelling<br>7- Refused<br>8- Dwelling not found<br>96- Other (Specify)............................
		**From**	**To**		
First visit	/ /				
Second visit	/ /				
Third visit	/ /				

Number of persons in household

Number of children (5-17)

Workers	Name	signature
supervisor.......... ☐☐	....	....
Interviewer........ ☐☐ ...:	....	....
Field editor......... ☐☐ ..:	....	....
Office editor...... ☐☐ ......:	....	....
Coder ☐		
Data entry.......... ☐☐ ....:	....	....
**If Additional Questionnaires used indicate additional number** ☐		

<table>
<tr><th>Section I:</th><th colspan="10">Household Composition and Characteristics for All Household Members</th></tr>
<tr>
<td rowspan="3">Person's serial number in household</td>
<td rowspan="3">Can you please provide full names of all persons who are part of this household, beginning with the Head of the Household?

(A Household is defined as a person or group of persons who live together in the same house or compound, share the same housekeeping arrangements and are catered for as one unit. Members of a household are not necessarily related (by blood or marriage) and not all those related in the same house or compound are necessarily of the same household)</td>
<td rowspan="3">Which household member provided information the individual (write serial number from A1)</td>
<td rowspan="3">What is (NAME)'s relationship to head of the household

1. Household Head
2. Spouse
3. Son / Daughter
4. Daughter-in-law / son-in-law
5. Grandchild
6. Father/ Mother
7. Brother/Sister
8. Father/ Mother in-law
9.Servant (live-in)
10. Other relative
11. Non-relative</td>
<td rowspan="3">What is the sex of each of these individual household members?

1. Male
2. Female</td>
<td rowspan="3">How old was (NAME) at (his/her) last birthday?

(In completed years)</td>
<td rowspan="3">Indicate

With "1" if person is between 5-17 years old, "0" otherwise</td>
<td rowspan="3">What is (NAME)'s marital status (for persons 18 years or above)

0 under age
1. Single or never married
2. Married contract
3. Married civil/religious
4. Divorced
5. Widowed
6. Married but separated</td>
<td colspan="3">For <u>all household members</u></td>
</tr>
<tr><td colspan="3">Please indicate (NAME) `s serial number .

(Write 95 if absent or not applicable)</td></tr>
<tr>
<td>Spouse

(if applicable and s/he is among the household members)</td>
<td>Natural Mother

(if she is among the household members)</td>
<td>Natural Father

(if he is among the household members)</td>
</tr>
<tr><th>A1</th><th>A2</th><th>A3</th><th>A4</th><th>A5</th><th>A6</th><th>A7</th><th>A8</th><th>A9</th><th>A10</th><th>A11</th></tr>
<tr><td>01</td><td></td><td>|__|__|</td><td>|__|__|</td><td>|__|</td><td>|__|__|</td><td>|__|</td><td>|__|</td><td>|__|__|</td><td>|__|__|</td><td>|__|__|</td></tr>
</table>

<table>
<tr><td>02</td><td></td><td>|__|__|</td><td>|__|__|</td><td>|__|</td><td>|__|__|</td><td>|__|</td><td>|__|</td><td>|__|__|</td><td>|__|__|</td><td>|__|__|</td></tr>
<tr><td>03</td><td></td><td>|__|__|</td><td>|__|__|</td><td>|__|</td><td>|__|__|</td><td>|__|</td><td>|__|</td><td>|__|__|</td><td>|__|__|</td><td>|__|__|</td></tr>
<tr><td>04</td><td></td><td>|__|__|</td><td>|__|__|</td><td>|__|</td><td>|__|__|</td><td>|__|</td><td>|__|</td><td>|__|__|</td><td>|__|__|</td><td>|__|__|</td></tr>
<tr><td>05</td><td></td><td>|__|__|</td><td>|__|__|</td><td>|__|</td><td>|__|__|</td><td>|__|</td><td>|__|</td><td>|__|__|</td><td>|__|__|</td><td>|__|__|</td></tr>
<tr><td>06</td><td></td><td>|__|__|</td><td>|__|__|</td><td>|__|</td><td>|__|__|</td><td>|__|</td><td>|__|</td><td>|__|__|</td><td>|__|__|</td><td>|__|__|</td></tr>
<tr><td>07</td><td></td><td>|__|__|</td><td>|__|__|</td><td>|__|</td><td>|__|__|</td><td>|__|</td><td>|__|</td><td>|__|__|</td><td>|__|__|</td><td>|__|__|</td></tr>
<tr><td>08</td><td></td><td>|__|__|</td><td>|__|__|</td><td>|__|</td><td>|__|__|</td><td>|__|</td><td>|__|</td><td>|__|__|</td><td>|__|__|</td><td>|__|__|</td></tr>
</table>

***IMPORTANT NOTE*:** **SECTION II onwards to be filled in column-wise beginning with the Serial No: 01 from A1**

**Section II:**	**Educational Attainment for All Household Members aged 5 and above**								
Serial No in A1	|__|__|	|__|__|	|__|__|	|__|__|	|__|__|	|__|__|	|__|__|	|__|__|	**Skip To Question**
Name of household member →									
Age of household member →	|__|__|	|__|__|	|__|__|	|__|__|	|__|__|	|__|__|	|__|__|	|__|__|	
1. **A12. Can (NAME) read and write a short, simple statement with understanding in any language?**<br>1. Yes................................<br>2. No................................	1<br>2	1<br>2	1<br>2	1<br>2	1<br>2	1<br>2	1<br>2	1<br>2	
2. **A13. Is (NAME) attending school or pre-school during the current school year?**<br>1. Yes................................<br>2. No................................	1<br>2	1<br>2	1<br>2	1<br>2	1<br>2	1<br>2	1<br>2	1<br>2	**→A14**<br>**→A15**

A14. What is the level of school and grade that (NAME) is attending?	L	G	L	G	L	G	L	G	L	G	L	G	L	G	L	G	
Level: (L) Grade: (G)																	
1. Pre-school*…………………	1	|__|	1	|__|	1	|__|	1	|__|	1	|__|	1	|__|	1	|__|	1	|__|	
2. Primary……………………	2	|__|	2	|__|	2	|__|	2	|__|	2	|__|	2	|__|	2	|__|	2	|__|	
3. preparatory…………………	3	|__|	3	|__|	3	|__|	3	|__|	3	|__|	3	|__|	3	|__|	3	|__|	
4. Secondary- general……….	4	|__|	4	|__|	4	|__|	4	|__|	4	|__|	4	|__|	4	|__|	4	|__|	
5. Secondary-Technical………	5	|__|	5	|__|	5	|__|	5	|__|	5	|__|	5	|__|	5	|__|	5	|__|	**A18**
6. Above intermediate………..	6	|__|	6	|__|	6	|__|	6	|__|	6	|__|	6	|__|	6	|__|	6	|__|	
7. University or higher………	7	|__|	7	|__|	7	|__|	7	|__|	7	|__|	7	|__|	7	|__|	7	|__|	
8. Non standard curriculum*…	**8**	|__|	**8**	|__|	**8**	|__|	**8**	|__|	**8**	|__|	**8**	|__|	**8**	|__|	**8**	|__|	
9. Don't now……………………	**9**	|__|	**9**	|__|	**9**	|__|	**9**	|__|	**9**	|__|	**9**	|__|	**9**	|__|	**9**	|__|	**A16**

put zero in grade in case of 1,8

Serial No in A1	|__|__|	|__|__|	|__|__|	|__|__|	|__|__|	|__|__|	|__|__|	|__|__|	Skip To Question
**Name of household member** →									
**Age of household member** →	|__|__|	|__|__|	|__|__|	|__|__|	|__|__|	|__|__|	|__|__|	|__|__|	

<table>
<tr><td>3. A15. Has (NAME) ever attended school?
1. Yes………………………..
2. No…………………………..</td><td colspan="2">1
2</td><td colspan="2">1
2</td><td colspan="2">1
2</td><td colspan="2">1
2</td><td colspan="2">1
2</td><td colspan="2">1
2</td><td colspan="2">1
2</td><td colspan="2">1
2</td><td>→A16
→A17</td></tr>
<tr><td rowspan="2">A16.What is the highest level of school and grade that (NAME) has attended and completed successfully?

Level: (L) Grade: (G)</td><td></td><td>G</td><td>L</td><td>G</td><td>L</td><td>G</td><td>L</td><td>G</td><td>L</td><td>G</td><td>L</td><td>G</td><td>L</td><td>G</td><td>L</td><td>G</td><td rowspan="11">}
A18</td></tr>
<tr><td></td><td></td><td></td><td></td><td></td><td></td><td></td><td></td><td></td><td></td><td></td><td></td><td></td><td></td><td></td><td></td></tr>
<tr><td>1. Pre-school*…………………</td><td>1</td><td>|__|</td><td>1</td><td>|__|</td><td>1</td><td>|__|</td><td>1</td><td>|__|</td><td>1</td><td>|__|</td><td>1</td><td>|__|</td><td>1</td><td>|__|</td><td>1</td><td>|__|</td></tr>
<tr><td>2. Primary……………………</td><td>2</td><td>|__|</td><td>2</td><td>|__|</td><td>2</td><td>|__|</td><td>2</td><td>|__|</td><td>2</td><td>|__|</td><td>2</td><td>|__|</td><td>2</td><td>|__|</td><td>2</td><td>|__|</td></tr>
<tr><td>3. preparatory…………………</td><td>3</td><td>|__|</td><td>3</td><td>|__|</td><td>3</td><td>|__|</td><td>3</td><td>|__|</td><td>3</td><td>|__|</td><td>3</td><td>|__|</td><td>3</td><td>|__|</td><td>3</td><td>|__|</td></tr>
<tr><td>4. Secondary- general……….</td><td>4</td><td>|__|</td><td>4</td><td>|__|</td><td>4</td><td>|__|</td><td>4</td><td>|__|</td><td>4</td><td>|__|</td><td>4</td><td>|__|</td><td>4</td><td>|__|</td><td>4</td><td>|__|</td></tr>
<tr><td>5. Secondary-Technical………</td><td>5</td><td>|__|</td><td>5</td><td>|__|</td><td>5</td><td>|__|</td><td>5</td><td>|__|</td><td>5</td><td>|__|</td><td>5</td><td>|__|</td><td>5</td><td>|__|</td><td>5</td><td>|__|</td></tr>
<tr><td>6. Above intermediate…………</td><td>6</td><td>|__|</td><td>6</td><td>|__|</td><td>6</td><td>|__|</td><td>6</td><td>|__|</td><td>6</td><td>|__|</td><td>6</td><td>|__|</td><td>6</td><td>|__|</td><td>6</td><td>|__|</td></tr>
<tr><td>7. University or higher………</td><td>7</td><td>|__|</td><td>7</td><td>|__|</td><td>7</td><td>|__|</td><td>7</td><td>|__|</td><td>7</td><td>|__|</td><td>7</td><td>|__|</td><td>7</td><td>|__|</td><td>7</td><td>|__|</td></tr>
<tr><td>8. Non standard curriculum*…</td><td>8</td><td>|__|</td><td>8</td><td>|__|</td><td>8</td><td>|__|</td><td>8</td><td>|__|</td><td>8</td><td>|__|</td><td>8</td><td>|__|</td><td>8</td><td>|__|</td><td>8</td><td>|__|</td></tr>
<tr><td>9. Don’t know</td><td>9</td><td>|__|</td><td>9</td><td>|__|</td><td>9</td><td>|__|</td><td>9</td><td>|__|</td><td>9</td><td>|__|</td><td>9</td><td>|__|</td><td>9</td><td>|__|</td><td>9</td><td>|__|</td></tr>
</table>

A17. What is/was the main reason why (NAME) has never attended school?<br><br>(Read each of the following options and circle the most appropriate option)									
1. Too young …………………..	01	01	01	01	01	01	01	01	}
2. Disabled/ illness………………	02	02	02	02	02	02	02	02	
3. No school/school too far…………………	03	03	03	03	03	03	03	03	
4. Cannot afford schooling………..	04	04	04	04	04	04	04	04	
5. Family did not allow schooling…	05	05	05	05	05	05	05	05	
6. Not interested in school…….	06	06	06	06	06	06	06	06	
7. Education not considered valuable.	07	07	07	07	07	07	07	07	
8. School not safe…………..	08	08	08	08	08	08	08	08	
9. To learn a job…………………	09	09	09	09	09	09	09	09	**A18**
10. To work for pay ……………	10	10	10	10	10	10	10	10	
11. To work as unpaid worker in family business/farm ……………………..	11	11	11	11	11	11	11	11	
12. Help at home with household chores……	12	12	12	12	12	12	12	12	
13. I haven't birth certificate	13	13	13	13	13	13	13	13	
96. Other…………………………..	96	96	96	96	96	96	96	96	
Other (specify)									

*** put zero in grade in case of 1,8**

Section III: Current Economic Activity Status of All Household Members (5 and above) during the reference week

									Skip To Question
Serial No in A1	|__|__|	|__|__|	|__|__|	|__|__|	|__|__|	|__|__|	|__|__|	|__|__|	
Name of household member →									
Age of household member →	|__|__|	|__|__|	|__|__|	|__|__|	|__|__|	|__|__|	|__|__|	|__|__|	
A. Employment									
A18 Did (NAME) engage in any work at least one hour during the past week?<br>***(As employee, self employed, employer or unpaid family worker)***<br>**1. Yes……………………..**<br>**2. No………………………**	1<br>2	1<br>2	1<br>2	1<br>2	1<br>2	1<br>2	1<br>2	1<br>2	**→A21**<br>**→A19**
**A19. During the past week did (NAME) do any of the following activities, even for only one hour?**<br>**(*Read each of the following questions until the first affrimative response is obtained*)**	**YES NO**	**YES NO**	**YES NO**	**YES NO**	**YES NO**	**YES NO**	**YES NO**	**YES NO**	
**(a) Run or do any kind of business, big or small, for himself/herself or with one or more partners?**<br>*Examples: Selling things, making things for sale, repairing things, guarding cars, hairdressing, crèche business, taxi or other transport business, having a legal or medical practice, performing in public, having a public phone shop, barber, shoe shining, production of ghee, butter, cheese etc.*	1 2	1 2	1 2	1 2	1 2	1 2	1 2	1 2	
**(b) Do any work for a wage, salary, commission or any payment in kind** (*excl. domestic work*)?<br>*Examples: a regular job, contract, casual or piece work for pay, work in exchange for food or housing working in quarries, tanneries.*	1 2	1 2	1 2	1 2	1 2	1 2	1 2	1 2	

**(c) Do any work as a domestic worker for a wage, salary or any payment in kind?**	1 2	1 2	1 2	1 2	1 2	1 2	1 2	1 2	
**(d) Help unpaid in a household business of any kind? (***Don't count normal housework***.)** *Examples: Help to sell things, make things for sale or exchange, doing the accounts, cleaning up for the business, etc.*	1 2	1 2	1 2	1 2	1 2	1 2	1 2	1 2	**If any "YES" → A21**
**(e) Do any work on his/her own or the household's plot, farm, food garden, or help in growing farm produce or in looking after animals for the household?** *Examples: ploughing, harvesting, looking after livestock.*	1 2	1 2	1 2	1 2	1 2	1 2	1 2	1 2	
**(f) Do any construction or major repair work on his/her own home, plot, or business or those of the household?**	1 2	1 2	1 2	1 2	1 2	1 2	1 2	1 2	**Otherwise**
**(g) Catch any fish, prawns, shells, wild animals or other food for sale or household food?**	1 2	1 2	1 2	1 2	1 2	1 2	1 2	1 2	**→A20**
**(h) Fetch water or collect firewood for household use?**	1 2	1 2	1 2	1 2	1 2	1 2	1 2	1 2	
**(i) Produce any other good for this household use?** *Examples: clothing, furniture, clay pots, etc.*	1 2	1 2	1 2	1 2	1 2	1 2	1 2	1 2	

Serial No in A1	|___|___|	|___|___|	|___|___|	|___|___|	|___|___|	|___|___|	|___|___|	|___|___|	**Skip To Question**
Name of household member →									
Age of household member →	|___|___|	|___|___|	|___|___|	|___|___|	|___|___|	|___|___|	|___|___|	|___|___|	
**A20. Even though (NAME) did not do any of these activities in the past week, does he/she have a job, business, or other economic or farming activity that he/she will definitely return to?** *(For agricultural activities, the off season in agriculture is not a temporary absence.)*									
1. Yes………………………..	1	1	1	1	1	1	1	1	→ **A21**
2. No…………………………..	2	2	2	2	2	2	2	2	→ **A33**
**A21. Describe the main job/task (NAME) was performing e.g. carrying bricks; mixing baking flour; harvesting maize; etc.** *("Main" refers to the work on which (NAME) spent most of the time during the week.)*									
**Job/Task**									
**OCCUPATION CODE** *For official use*	|___|___|___|	|___|___|___|	|___|___|___|	|___|___|___|	|___|___|___|	|___|___|___|	|___|___|___|	|___|___|___|	
A22. Describe briefly the main activity i.e. goods produced and services rendered where (NAME) is working.									
**Activity/Type**									
**INDUSTRY CODE** *For official use*	|__|__|__|__|	|__|__|__|__|	|__|__|__|__|	|__|__|__|__|	|__|__|__|__|	|__|__|__|__|	|__|__|__|__|	|__|__|__|__|	

A23. Where did (NAME) carry out his/her main work during the past week? *(Read out responses below)*									
1. At (his/her) family dwelling…	01	01	01	01	01	01	01	01	
2. At dweling for other………….	02	02	02	02	02	02	02	02	
3. Client`s place ……………….	03	03	03	03	03	03	03	03	
4. Formal office …………….	04	04	04	04	04	04	04	04	
5. Factory / Atelier ………..	05	05	05	05	05	05	05	05	
6. Plantations / farm / garden…………….	06	06	06	06	06	06	06	06	
7. Construction sites…………….	07	07	07	07	07	07	07	07	
8. Mines / quarry………….	08	08	08	08	08	08	08	08	
9. Shop / kiosk / coffee house / restaurant / hotel	09	09	09	09	09	09	09	09	
10. Different places (mobile)………….	10	10	10	10	10	10	10	10	
11. Fixed market stall…………….	11	11	11	11	11	11	11	11	
12. In street…………….	12	12	12	12	12	12	12	12	
13. Pond/lake/river…………………………….	13	13	13	13	13	13	13	13	
96. Other………………………………	96	96	96	96	96	96	96	96	
Other (specify)									
**A24. During the past week, which of the following best describe (NAME) `s work situation at his/her main work?** *(Read out responses below)*									
1. Employee……….	1	1	1	1	1	1	1	1	**→A25**
2. Own account worker (His/her own business without employees) ……..	2	2	2	2	2	2	2	2	
3. Employer (His/her own business with employees)	3	3	3	3	3	3	3	3	} **A28**
4. Unpaid family worker……	4	4	4	4	4	4	4	4	**→ A30**

Serial No in A1	|___|___|	|___|___|	|___|___|	|___|___|	|___|___|	|___|___|	|___|___|	|___|___|	**Skip To Question**
**Name of household member** →									
**Age of household member** →	|___|___|	|___|___|	|___|___|	|___|___|	|___|___|	|___|___|	|___|___|	|___|___|	
**A25. Has (NAME) been employed on the basis of**									
1. A written contract...	1	1	1	1	1	1	1	1	
2. A verbal agreement...........	2	2	2	2	2	2	2	2	
8. Don`t know...	8	8	8	8	8	8	8	8	
**A26. Is (NAME)`s contract/agreement……**									
1. Limited duration...	1	1	1	1	1	1	1	1	**→A27**
2. Unlimited duration............	2	2	2	2	2	2	2	2	} **A28**
8. Don`t know...	8	8	8	8	8	8	8	8	} **A28**
**A27. What is the duration of (NAME)`s contract/ agreement?**									
1. Less than 12 months...........................	1	1	1	1	1	1	1	1	
2. 12-36 months............................	2	2	2	2	2	2	2	2	
3. More than 36 months	3	3	3	3	3	3	3	3	
8. Don't know...	8	8	8	8	8	8	8	8	

**A28. What is (Name's) average monthly cash income from the main work? (*in local currency*)**	|__|__|__|__|	|__|__|__|__|	|__|__|__|__|	|__|__|__|__|	|__|__|__|__|	|__|__|__|__|	|__|__|__|__|	|__|__|__|__|	
**Check A24! if A24=2 or 3 then skip to A30**									
**A29. What other benefits does (NAME) usually receive in his/her main work?**<br><br>**(***Read each of the following questions and circle answers. Multiple answers are allowed***)**									
A. Weekly rest days….	A	A	A	A	A	A	A	A	
B. Medical expenses………	B	B	B	B	B	B	B	B	
C. School expenses……	C	C	C	C	C	C	C	C	
D. Assistance with schooling …	D	D	D	D	D	D	D	D	
E. Paid sick leave……	E	E	E	E	E	E	E	E	
F. Annual vacation…..	F	F	F	F	F	F	F	F	
G. Free/subsidized accommodation………..	G	G	G	G	G	G	G	G	
H. Food/meal…………………	H	H	H	H	H	H	H	H	
I. Paid leave …….	I	I	I	I	I	I	I	I	
J. Clothing…………..………	J	J	J	J	J	J	J	J	
K. Transportation ………….	K	K	K	K	K	K	K	K	
U. Other ………..	U	U	U	U	U	U	U	U	
X. Don't know	X	X	X	X	X	X	X	X	
Y. Nothing……	Y	Y	Y	Y	Y	Y	Y	Y	
**Other (**specify**)**									

<table>
<tr><td>Serial No in A1</td><td colspan="2">|___|___|</td><td colspan="2">|___|___|</td><td colspan="2">|___|___|</td><td colspan="2">|___|___|</td><td colspan="2">|___|___|</td><td colspan="2">|___|___|</td><td colspan="2">|___|___|</td><td colspan="2">|___|___|</td><td rowspan="3">Skip
To
Question</td></tr>
<tr><td>Name of household member →</td><td colspan="2"></td><td colspan="2"></td><td colspan="2"></td><td colspan="2"></td><td colspan="2"></td><td colspan="2"></td><td colspan="2"></td><td colspan="2"></td></tr>
<tr><td>Age of household member →</td><td colspan="2">|___|___|</td><td colspan="2">|___|___|</td><td colspan="2">|___|___|</td><td colspan="2">|___|___|</td><td colspan="2">|___|___|</td><td colspan="2">|___|___|</td><td colspan="2">|___|___|</td><td colspan="2">|___|___|</td></tr>
<tr><td>A30. In addition to (NAME)'s main work, did (NAME) do any other work during the past week?
1. Yes………………………………
2. No………………………………</td><td colspan="2">1
2</td><td colspan="2">1
2</td><td colspan="2">1
2</td><td colspan="2">1
2</td><td colspan="2">1
2</td><td colspan="2">1
2</td><td colspan="2">1
2</td><td colspan="2">1
2</td><td></td></tr>
<tr><td>A31. For each day worked during the past week how many hours did (NAME) actually work?</td><td>M</td><td>O</td><td>M</td><td>O</td><td>M</td><td>O</td><td>M</td><td>O</td><td>M</td><td>O</td><td>M</td><td>O</td><td>M</td><td>O</td><td>M</td><td>O</td><td></td></tr>
<tr><td>Main: (M) Other: (O)</td><td></td><td></td><td></td><td></td><td></td><td></td><td></td><td></td><td></td><td></td><td></td><td></td><td></td><td></td><td></td><td></td><td></td></tr>
<tr><td>1. Monday……………………</td><td>|__|__|</td><td>|__|</td><td>|__|__|</td><td>|__|</td><td>|__|__|</td><td>|__|</td><td>|__|__|</td><td>|__|</td><td>|__|__|</td><td>|__|</td><td>|__|__|</td><td>|__|</td><td>|__|__|</td><td>|__|</td><td>|__|__|</td><td>|__|</td><td></td></tr>
<tr><td>2. Tuesday……………………</td><td>|__|__|</td><td>|__|</td><td>|__|__|</td><td>|__|</td><td>|__|__|</td><td>|__|</td><td>|__|__|</td><td>|__|</td><td>|__|__|</td><td>|__|</td><td>|__|__|</td><td>|__|</td><td>|__|__|</td><td>|__|</td><td>|__|__|</td><td>|__|</td><td></td></tr>
<tr><td>3. Wednesday………………</td><td>|__|__|</td><td>|__|</td><td>|__|__|</td><td>|__|</td><td>|__|__|</td><td>|__|</td><td>|__|__|</td><td>|__|</td><td>|__|__|</td><td>|__|</td><td>|__|__|</td><td>|__|</td><td>|__|__|</td><td>|__|</td><td>|__|__|</td><td>|__|</td><td></td></tr>
<tr><td>4. Thursday…………………</td><td>|__|__|</td><td>|__|</td><td>|__|__|</td><td>|__|</td><td>|__|__|</td><td>|__|</td><td>|__|__|</td><td>|__|</td><td>|__|__|</td><td>|__|</td><td>|__|__|</td><td>|__|</td><td>|__|__|</td><td>|__|</td><td>|__|__|</td><td>|__|</td><td></td></tr>
<tr><td>5. Friday………………………</td><td>|__|__|</td><td>|__|</td><td>|__|__|</td><td>|__|</td><td>|__|__|</td><td>|__|</td><td>|__|__|</td><td>|__|</td><td>|__|__|</td><td>|__|</td><td>|__|__|</td><td>|__|</td><td>|__|__|</td><td>|__|</td><td>|__|__|</td><td>|__|</td><td></td></tr>
<tr><td>6. Saturday…………………..</td><td>|__|__|</td><td>|__|</td><td>|__|__|</td><td>|__|</td><td>|__|__|</td><td>|__|</td><td>|__|__|</td><td>|__|</td><td>|__|__|</td><td>|__|</td><td>|__|__|</td><td>|__|</td><td>|__|__|</td><td>|__|</td><td>|__|__|</td><td>|__|</td><td></td></tr>
<tr><td>7. Sunday………………………</td><td>|__|__|</td><td>|__</td><td>|__|__|</td><td>|__|</td><td>|__|__|</td><td>|__|</td><td>|__|__|</td><td>|__|</td><td>|__|__|</td><td>|__|</td><td>|__|__|</td><td>|__|</td><td>|__|__|</td><td>|__|</td><td>|__|__|</td><td>|__|</td><td></td></tr>
</table>

**TOTAL**	|__|__|	|__|__|	|__|__|	|__|__|	|__|__|	|__|__|	|__|__|	|__|__|	
**A32. At what age did (NAME) start to work for the first time in his/her life** (*As employee, own account worker, employed, employer or unpaid family worker*)?	|__|__|	|__|__|	|__|__|	|__|__|	|__|__|	|__|__|	|__|__|	|__|__|	**→A40**

**Serial No in A1**	|___|___|	|___|___|	|___|___|	|___|___|	|___|___|	|___|___|	|___|___|	|___|___|	**Skip To Question**
**Name of household member** →									
**Age of household member** →	|___|___|	|___|___|	|___|___|	|___|___|	|___|___|	|___|___|	|___|___|	|___|___|	

B. Unemployment									Aged 5-9 years	Aged 10 years and over
**A33. Was (NAME) seeking work during the past week?** (*As employee, employer or own-account worker to establish his/her own business*)<br>1. Yes………………………<br>2. No……………….……….	1<br>2	1<br>2	1<br>2	1<br>2	1<br>2	1<br>2	1<br>2	1<br>2	A37	→A34<br>→A35
**A34. What steps did (NAME) take during the past four weeks to find work? (***Mark at most 4 boxes)*<br>A. Asked friend or relatives to find a job for him/her.<br>B. Applied to the employment office/mediator<br>C. Placed/answered job advertisements in newspaper<br>D. Submitted job application ……<br>E. Tried to obtain equipment, credit and/or a work place to establish his/her own business<br>U. Other ……………<br>X. Don't know…………<br>Y Nothing………………..	A<br>B<br>C<br>D<br>E<br>U<br>X<br>Y	A<br>B<br>C<br>D<br>E<br>U<br>X<br>Y	A<br>B<br>C<br>D<br>E<br>U<br>X<br>Y	A<br>B<br>C<br>D<br>E<br>U<br>X<br>Y	A<br>B<br>C<br>D<br>E<br>U<br>X<br>Y	A<br>B<br>C<br>D<br>E<br>U<br>X<br>Y	A<br>B<br>C<br>D<br>E<br>U<br>X<br>Y	A<br>B<br>C<br>D<br>E<br>U<br>X<br>Y		A37<br>→A35
**Other** (specify)										
**A35. Did (NAME) want to work during the past week?**<br>1. Yes……………………….<br>2. No……………………….	1<br>2	1<br>2	1<br>2	1<br>2	1<br>2	1<br>2	1<br>2	1<br>2		→A36<br>→A39
**A36. What is the main reason why (NAME) did**										

not seek work during the past week? (*Indicate the most important reason*)										
1. Found a job but waiting to start	01	01	01	01	01	01	01	01		
2. Works seasonally ………	02	02	02	02	02	02	02	02		
3. Tired of looking for work, believes no suitable work is available….	03	03	03	03	03	03	03	03		
4. Lacks employers` requirements (training, experience, qualification)	04	04	04	04	04	04	04	04		
5. Does not know where to search for a job	05	05	05	05	05	05	05	05		
6. Student (studying)…..	06	06	06	06	06	06	06	06		
7. Family/parents/spouse does not allow…..	07	07	07	07	07	07	07	07		
8. Engaged in household chores………	08	08	08	08	08	08	08	08		
9. Unable to work (illness, disability)	09	09	09	09	09	09	09	09		
10. Waiting hiring	10	10	10	10	10	10	10	10		
11. prefer male than female in hiring	11	11	11	11	11	11	11	11		
12. prefer female than male in hiring	12	12	12	12	12	12	12	12		
13. No social relations to help me to find chance in hiring	13	13	13	13	13	13	13	13		
14. Too young for work........	14	14	14	14	14	14	14	14		
96. Other…………..…	96	96	96	96	96	96	96	96		
**Other (**specify**)**										

**Serial No in A1**	I___I___I	I___I___I	I___I___I	I___I___I	I___I___I	I___I___I	I___I___I	I___I___I	**Skip to Question**	
**Name of household member** →									**Aged 5-9 years**	**Aged 10 years and over**
**Age of household member** →	I___I___I	I___I___I	I___I___I	I___I___I	I___I___I	I___I___I	I___I___I	I___I___I		

A37. If opportunity to work had existed, would (NAME) have been able to start work in the past week?										
1. Yes……………………	1	1	1	1	1	1	1	1	A41	→A38
2. No……………………..	2	2	2	2	2	2	2	2		→A39
**A38. How long has (NAME) been out of work and seeking work?**										
1. Less than one month….	1	1	1	1	1	1	1	1		A41
2. 1 to 3 month…………	2	2	2	2	2	2	2	2		
3. 4 to 6 month……….	3	3	3	3	3	3	3	3		
4. 7 to 12 month……….	4	4	4	4	4	4	4	4		
5. 13 to 24 months……….	5	5	5	5	5	5	5	5		
6. More than 2 years….	6	6	6	6	6	6	6	6		
**A39. Why was (NAME) not available or did not want to work? (Indicate the most important reason)**										
1. Found a job but waiting to start	01	01	01	01	01	01	01	01		
2. Works seasonally ………	02	02	02	02	02	02	02	02		A41
3. Tired of looking for work, believes no suitable work is available….	03	03	03	03	03	03	03	03		
4. Lack of employers` requirement (training, experience, qualification)	04	04	04	04	04	04	04	04		
5. Does not know where to search for a job..	05	05	05	05	05	05	05	05		
6. Student (studying)…..	06	06	06	06	06	06	06	06		
7. Family/parents/spouse does not allow…..	07	07	07	07	07	07	07	07		
8. Engaged in household chores ………	08	08	08	08	08	08	08	08		
9. Unable to work (illness, disability)	09	09	09	09	09	09	09	09		
10. Waiting hiring	10	10	10	10	10	10	10	10		
11. prefer male than female in hiring	11	11	11	11	11	11	11	11		
12. prefer female than male in hiring	12	12	12	12	12	12	12	12		
13. No social relations to help me to find chance in hiring	13	13	13	13	13	13	13	13		

14. Too young for work........<br>96. Other……………..	14<br>96	14<br>96	14<br>96	14<br>96	14<br>96	14<br>96	14<br>96	14<br>96		
**Other (**specify**)**										

Section IV: Usual Employment Status of All Household Members (5 and above) during the last 12 months

									Skip To Question
Serial No in A1	I___I___I	I___I___I	I___I___I	I___I___I	I___I___I	I___I___I	I___I___I	I___I___I	
Name of household member →									
Age of household member →	I___I___I	I___I___I	I___I___I	I___I___I	I___I___I	I___I___I	I___I___I	I___I___I	
A40. Was the work reported in A21, A22 and A24 (NAME)`s main employment during the past 12 months? ***(As employee, own account worker, employer or unpaid family worker)***									
1. Yes……………………	1	1	1	1	1	1	1	1	→ A46
**2. No……………………..**	2	2	2	2	2	2	2	2	→ A43
A 41 Did (NAME) engage in any work at least one hour during the past 12 months? ***(As employee, self employed, employer or unpaid family worker)***									
**1. Yes……………………**	1	1	1	1	1	1	1	1	→A43
2. No……………………..	2	2	2	2	2	2	2	2	→A42
A 42. In the past twelve months, did (NAME) do any of the following activities, even for only one hour? (***Read each of the following questions until the first affirmative response is obtained***)	**YES NO**	**YES NO**	**YES NO**	**YES NO**	**YES NO**	**YES NO**	**YES NO**	**YES NO**	
(a) **Run or do any kind of business, big or small, for himself/herself or with one or more partners?** *Examples: Selling things, making things for sale, repairing things, guarding cars, hairdressing, crèche business, taxi or other transport business, having a legal or medical practice, performing in public, having a public phone shop, barber, shoe shining, , production of ghee, butter, cheese etc.*	1 2	1 2	1 2	1 2	1 2	1 2	1 2	1 2	
(b) **Do any work for a wage, salary, commission or any payment in kind** (*excl. domestic work*)**?** *Examples: a regular job, contract, casual or piece work for pay, work in exchange for food or housing. working in quarries, tanneries*	1 2	1 2	1 2	1 2	1 2	1 2	1 2	1 2	

(c) **Do any work as a domestic worker for a wage, salary or any payment in kind?**	1 2	1 2	1 2	1 2	1 2	1 2	1 2	1 2	
(d) **Help unpaid in a household business of any kind?** *(Don't count normal housework.)*<br>*Examples: Help to sell things, make things for sale or exchange, doing the accounts, cleaning up for the business, etc,*	1 2	1 2	1 2	1 2	1 2	1 2	1 2	1 2	**If any**
(e) **Do any work on his/her own or the household's plot, farm, food garden, or help in growing farm produce or in looking after animals for the household?**<br>*Examples: ploughing, harvesting, looking after livestock.*	1 2	1 2	1 2	1 2	1 2	1 2	1 2	1 2	**"YES"**<br>**→ A43**
(f) **Do any construction or major repair work on his/her own home, plot, or business or those of the household?**	1 2	1 2	1 2	1 2	1 2	1 2	1 2	1 2	
(g) **Catch any fish, prawns, shells, wild animals or other food for sale or household food?**	1 2	1 2	1 2	1 2	1 2	1 2	1 2	1 2	**Otherwise**<br>If Age <18→**A47**
(h) **Fetch water or collect firewood for household use?**	1 2	1 2	1 2	1 2	1 2	1 2	1 2	1 2	If Age ≥18→**END**
(i) **Produce any other good for this household use?**<br>*Examples: clothing, furniture, clay pots, etc.*	1 2	1 2	1 2	1 2	1 2	1 2	1 2	1 2	for this HH member.<br>Go to the next HH member in Section II

<table>
<tr><td>Serial No in A1</td><td>|___|___|</td><td>|___|___|</td><td>|___|___|</td><td>|___|___|</td><td>|___|___|</td><td>|___|___|</td><td>|___|___|</td><td>|___|___|</td><td rowspan="3">Skip
To
Question</td></tr>
<tr><td>Name of household member →</td><td></td><td></td><td></td><td></td><td></td><td></td><td></td><td></td></tr>
<tr><td>Age of household member →</td><td>|___|___|</td><td>|___|___|</td><td>|___|___|</td><td>|___|___|</td><td>|___|___|</td><td>|___|___|</td><td>|___|___|</td><td>|___|___|</td></tr>
<tr><td colspan="9">A43. Describe the main job/task (NAME) was performing during the last 12 months e.g. carrying bricks; mixing baking flour; harvesting maize; etc.
(“Main” refers to the work on which (NAME) spent most of the time during the year.)</td><td></td></tr>
<tr><td>Job/Task</td><td></td><td></td><td></td><td></td><td></td><td></td><td></td><td></td><td></td></tr>
<tr><td>OCCUPATION CODE
For official use</td><td>|___|___|___|</td><td>|___|___|___|</td><td>|___|___|___|</td><td>|___|___|___|</td><td>|___|___|___|</td><td>|___|___|___|</td><td>|___|___|___|</td><td>|___|___|___|</td><td></td></tr>
<tr><td colspan="9">A44. Describe briefly the main goods produced and /or services rendered where (NAME) worked most of the time.</td><td></td></tr>
<tr><td>Activity/Type</td><td></td><td></td><td></td><td></td><td></td><td></td><td></td><td></td><td></td></tr>
<tr><td>INDUSTRY CODE
For official use</td><td>|__|__|__|__|</td><td>|__|__|__|__|</td><td>|__|__|__|__|</td><td>|__|__|__|__|</td><td>|__|__|__|__|</td><td>|__|__|__|__|</td><td>|__|__|__|__|</td><td>|__|__|__|__|</td><td></td></tr>
<tr><td>A45. Which of the following best describe (NAME) `s work situation at his/her main work in the past 12 months? (Read out responses below)
1. Employee………………..
2. Own account worker (His/her own business without employees)…
3. Employer (His/her own business with employees)
4. Unpaid family worker………..</td><td>1
2
3
4</td><td>1
2
3
4</td><td>1
2
3
4</td><td>1
2
3
4</td><td>1
2
3
4</td><td>1
2
3
4</td><td>1
2
3
4</td><td>1
2
3
4</td><td></td></tr>
</table>

**A46. In each month during the past year did (NAME) work or have a job?**	**1= YES 2=NO**	**1= YES 2=NO**	**1= YES 2=NO**	**1= YES 2=NO**	**1= YES 2=NO**	**1= YES 2=NO**	**1= YES 2=NO**	**1= YES 2=NO**	
(M*ark "YES" or "NO" for all months)*									
1. January…………………	01|___|	01|___|	01|___|	01|___|	01|___|	01|___|	01|___|	01|___|	If Age <18→**A47**
2. February………………	02|___|	02|___|	02|___|	02|___|	02|___|	02|___|	02|___|	02|___|	
3. March……………………	03|___|	03|___|	03|___|	03|___|	03|___|	03|___|	03|___|	03|___|	
4. April………………………	04|___|	04|___|	04|___|	04|___|	04|___|	04|___|	04|___|	04|___|	
5. May…………………….	05|___|	05|___|	05|___|	05|___|	05|___|	05|___|	05|___|	05|___|	**<u>Otherwise</u>**
6. June…………………….	06|___|	06|___|	06|___|	06|___|	06|___|	06|___|	06|___|	06|___|	**END**
7. July………………………	07|___|	07|___|	07|___|	07|___|	07|___|	07|___|	07|___|	07|___|	
8. August……………………	08|___|	08|___|	08|___|	08|___|	08|___|	08|___|	08|___|	08|___|	for this HH member.
9. September…………..	09|___|	09|___|	09|___|	09|___|	09|___|	09|___|	09|___|	09|___|	
10. October…………………	10|___|	10|___|	10|___|	10|___|	10|___|	10|___|	10|___|	10|___|	Go to the next HH member in Section II
11. November…………..	11|___|	11|___|	11|___|	11|___|	11|___|	11|___|	11|___|	11|___|	
12. December…………….	12|___|	12|___|	12|___|	12|___|	12|___|	12|___|	12|___|	12|___|	
**TOTAL**	|___|___|	|___|___|	|___|___|	|___|___|	|___|___|	|___|___|	|___|___|	|___|___|	

Section V: Household Tasks: About Children (5-17) ONLY

Serial No in A1	|___|___|	|___|___|	|___|___|	|___|___|	|___|___|	|___|___|	|___|___|	|___|___|	Skip To Question
Name of household member →									
Age of household member →	|___|___|	|___|___|	|___|___|	|___|___|	|___|___|	|___|___|	|___|___|	|___|___|	
**A47. During the past week did (NAME) do any of the tasks indicated below for this household?** (*Read each of the following options and mark "YES" or "NO" for all options*)	1= YES 2=NO	1= YES 2=NO	1= YES 2=NO	1= YES 2=NO	1= YES 2=NO	1= YES 2=NO	1= YES 2=NO	1= YES 2=NO	If any **"YES"** **→A48**
1. Shopping for household....	1|___|	1|___|	1|___|	1|___|	1|___|	1|___|	1|___|	1|___|	If all **"NO"** **&**Working **(*)→A49**
2. Repairing any household equipment	2|___|	2|___|	2|___|	2|___|	2|___|	2|___|	2|___|	2|___|	
3. Cooking..	3|___|	3|___|	3|___|	3|___|	3|___|	3|___|	3|___|	3|___|	**Otherwise**
4. Cleaning utensils/house.................	4|___|	4|___|	4|___|	4|___|	4|___|	4|___|	4|___|	4|___|	
5. Washing clothes..........................	5|___|	5|___|	5|___|	5|___|	5|___|	5|___|	5|___|	5|___|	**END** for this HH Member.
6. Caring for children/old/sick..........	6|___|	6|___|	6|___|	6|___|	6|___|	6|___|	6|___|	6|___|	
7. Other household tasks.....	7|___|	7|___|	7|___|	7|___|	7|___|	7|___|	7|___|	7|___|	Go to the next HH member in Section II
**Other** (specify)									

A48.During each day of the past weekhow many hours did (NAME) do this household task?<br><br>*(Record for each day separately)*									
1. Monday………………………	|__|	|__|	|__|	|__|	|__|	|__|	|__|	|__|	
2. Tuesday………………………	|__|	|__|	|__|	|__|	|__|	|__|	|__|	|__|	If Working **(*)**
3. Wednesday……………………	|__|	|__|	|__|	|__|	|__|	|__|	|__|	|__|	**→A49**
4. Thursday……………………	|__|	|__|	|__|	|__|	|__|	|__|	|__|	|__|	
5. Friday…………………………	|__|	|__|	|__|	|__|	|__|	|__|	|__|	|__|	**<u>Otherwise</u>**
6. Saturday……………………	|__|	|__|	|__|	|__|	|__|	|__|	|__|	|__|	**END** for this HH Member.
7. Sunday…………………….	|__|	|__|	|__|	|__|	|__|	|__|	|__|	|__|	
**TOTAL**	|__|__|	|__|__|	|__|__|	|__|__|	|__|__|	|__|__|	|__|__|	|__|__|	Go to the next HH member in Section II

(*)WORKING = IF A18=*YES* or A19=*YES* or A20=*YES*

Attention: **Section VI applies ONLY to those working (A18=YES or A19=YES or A20=YES) children age 5-17 (A7=1).**

Section VI

1. Perceptions/Observations of Parents/Guardians about working children (5-17)

These questions are intended to solicit views from parents or guardians about children's work.. Therefore reference should only be made about children who were reported to be working.

									Skip To Question
**Serial No in A1**	I___I___I	I___I___I	I___I___I	I___I___I	I___I___I	I___I___I	I___I___I	I___I___I	
**Name of household member** →									
**Age of household member** →	I___I___I	I___I___I	I___I___I	I___I___I	I___I___I	I___I___I	I___I___I	I___I___I	
**A 49. What do you consider currently best for (NAME)?***(Read the options)*									
1. Work for income………	1	1	1	1	1	1	1	1	
2. to gain a vocation	2	2	2	2	2	2	2	2	
3. Assist family business……	3	3	3	3	3	3	3	3	
4. Assist with household chores	4	4	4	4	4	4	4	4	
5. Attend school ……….	5	5	5	5	5	5	5	5	
7. Other …………………………	7	7	7	7	7	7	7	7	
**Other** (specify)									

A 50. What problem(s) does (NAME) face as a result of his/her work?) ***(Read the options and circle all the ones that are appropriate.)***									
A. Injury, illness or poor health……	A	A	A	A	A	A	A	A	
B. Poor grades in school………	B	B	B	B	B	B	B	B	
C. Emotional harassment (intimidation, scolding, insulting)………………….	C	C	C	C	C	C	C	C	
D. Physical harassment (beating)…	D	D	D	D	D	D	D	D	
E. Sexual abuse………………	E	E	E	E	E	E	E	E	
F. Extreme fatigue……………………	F	F	F	F	F	F	F	F	
G. No play time…………………	G	G	G	G	G	G	G	G	
H. No time to go to school………..	H	H	H	H	H	H	H	H	
I. No time for the homework or to review lessons	I	I	I	I	I	I	I	I	
J. None………………………………………….	J	J	J	J	J	J	J	J	
U. Others ……………………	U	U	U	U	U	U	U	U	
A51. What are the main reasons for letting (NAME) work? ***(Indicate three most important reasons)***									Go to the next HH member in Section II
A. Supplement family income………	A	A	A	A	A	A	A	A	
B. Help pay family debt………..	B	B	B	B	B	B	B	B	
C. Help in household enterprise…	C	C	C	C	C	C	C	C	
D. Learn skills……………………	D	D	D	D	D	D	D	D	
E. Schooling not useful for future…	E	E	E	E	E	E	E	E	
F. No school/school too far…………………	F	F	F	F	F	F	F	F	
G. Cannot afford school fees or other expeses for education	G	G	G	G	G	G	G	G	
H. Child not interested in school……	H	H	H	H	H	H	H	H	
I. Temporarily replacing someone unable to work.	I	I	I	I	I	I	I	I	
J. Preventing him/her from making bad friends and/or being led astray	J	J	J	J	J	J	J	J	
U. Other…………………………………………….	U	U	U	U	U	U	U	U	

Other (specify)									

Go to the 2nd part of the Questionnaire to ask questions on the household characteristics

PART II HOUSEHOLD CHARACTERISTICS

Addressed to the most knowledgeable member of household **HOUSEHOLD ID NUMBER :**

SECTION VII Housing and Household Characteristics

10 JAN 2008

**B1. In what type of dwelling does the household live?**		**Skip to question**
1. Apartment/flat	1	
2. More than one apartment	2	
3. Villa or Private house	3	
4. Countryside house	4	
5. Part of a private house (room or more in a house unit)	5	
6. Separate room or more	6	
7. Mobile home (e.g. tent, caravan).	7	
8. Shelter not meant for living purposes (burial Hall)	8	
9. Other…………………	9	
**Other***(specify)*		

**B2. What is the ownership status of this dwelling?**		
1. Rented (ordinary low rent)	1	
2. Rented (new low rent)	2	
3. Rented (furniture rent)	3	
4. Owned by any household member	4	
5. Donation	5	
6. Provided free(gift)	6	
7. Subsidised by employer (lodging)……	7	
87. Other…	8	
**Other** *(specify)*		

**B3. How many rooms are there in this dwelling** (include hall)**?**	|___|___|	

**B4. What is the size of dwelling in square metres?**		
1. Less than 20 square metres…	1	
2. 20 to 39 square metres………	2	
3. 40 to 69 square metres………	3	
4. 70 to 99 square metres……….	4	
5. 100 square metres or more…….	5	

**B5. Are any of these facilities available to the household? *(Enter appropriate code for each facility)***	KITCHEN	BATHROOM	TOILET	
		1	1	
1. Inside house and exclusive…..	1	2	2	
2. Inside house and shared……	2	3	3	
3. Outside house and exclusive…	3	4	4	
4. Outside house and shared…	4	95	95	
95. Not available…………..	95			

**B6. What is the main source of energy?**	**COOKING**	**HEATING**	**COOLING**	**LIGHTING**	
0. Nothing………..	00	00	00	00	
1. Gas bottle ………..	01	01	01	01	
2. Natural Gas……………	02	02	02	02	
3. Kerosene……….	03	03	03	03	
4. Electricity………	04	04	04	04	
96. Other…	96	96	96	96	

**Other** *(Specify)*				

**B7. What is the main source of drinking water?**		**Skip to question**
01. Pipe-borne inside house……	01	
02. Pipe-borne outside house….	02	
03. River/stream……………	03	
04. Bore-hole/tube well ….	04	
05. Public Tap	05	
06. Tanker service ……….	06	
96. Other……..	96	
**Other** ***(Specify)***		
B8. Has the household ever changed the place of residence? ***(Kism/MarkzLGovernorate/country)***		
1. Yes……………………..	1	→B9
2. No……………………	2	→B12

4. **B9. In which district/province/country was the last place of residence of the household?**	----------------------------	**CODES** ***(For official use)***	
Governorate:	…………………………………….	|___|___|	
**Urban/Rural:**	…………………………………	|___|	
**City/kism/markz**	…………………………………	|___|___||___|	
**Country:**		|___|___||___|	

**B10. In which year did the household move to the present place of residence?**	|___|___|___|___|	
B11. What was the main reason for coming or changing to the present place of residence?		
01 Job transfer………………..	01	
02 Found a job………………………	02	
03 Looking for job…………………..	03	
04 Looking for better agricultural land…	04	
05 Studies (Schooling/training)……….	05	
06 Proximity to place of work	06	
07 Looking for better housing	07	
08 Looking for more affordable housing	08	
09 Social problem	09	
10 political problem	10	
11 Health ……………..	11	
96 Other …………………….	96	
**Other***(Specify)*		

SECTION VIII Household Socio-Economic Status

B12. Does the household own any of the following? (*Mark "YES" or "NO" for all options)*	YES	NO	Skip to question
1. Air condaition	1	2	
2. Air heat	1	2	
3. Heater	1	2	
4. Automobile…………	1	2	
5. Tractor………..	1	2	
6. Motor-bike…………	1	2	
7. Toktok	1	2	
8. Bicycle………………..	1	2	
9. Animal drawn-cart…	1	2	
10. Television………….	1	2	
11. Iron………….	1	2	
12. VCD/DVD player……	1	2	
13. Washing machine aoutomatic……	1	2	
14. Washing machine……	1	2	
15. Oven(potagaz)…………………	1	2	
16. Dishwasher………….	1	2	
17. Refrigerator…………	1	2	
18. Computer/Laptop…………	1	2	
19. Sewing machine………	1	2	
20. Satellite/Cable TV…….	1	2	
21. Telephone (Land line)…	1	2	
22. Mobile phone…………	1	2	
23. Radio/casit…………..	1	2	

B13. Does the household own any livestock?		
1. Yes……………………	1	→B14
2. No…………………	2	→B15
**B14. How many?**	*In number*	
1. Camel……………………	|__|__|__|	
2. Horse/donky…………………	|__|__|__|	
3. Cow/buffalo………………	|__|__|__|	
4. Sheep……………………..	|__|__|__|	
5. Goat………………………	|__|__|__|	
6. Poultry…………………….	|__|__|__|	
96 Other……………………..	|__|__|__|	
**B15. Does the household own any land?**		
1. Yes……………………….	1	→B16
2. No………………………..	2	→B17
**B16. How many ares of land does the household own?**		
(1 are =100 square meters)	Ares	
1. Agricultural (cultivable)….	|__|__|__|	
7. Other …………………...	|__|__|__|	
**Other** (*Specify*)		

**B17.Has the household been adversely affected by any (*countrywide/communitywide*) problem in the last 12 months?**		**Skip to question**
1. Yes……………………	1	**→B18**
2. No…………………	2	**→B19**

**B18. What was the problem?** (*Indicate the most important faced*)		
1. Natural disaster (drought, flood, storms, hurricane, landslides, forest fires)………………	1	
2. Epidemics…………………	2	
3. Business closing due to economic recession	3	
4. Falling agricultural prices.	4	
5. Price inflation …………..	5	
6. Public protests ………….	6	
7. Other ……………………….	7	
**Other** ***(Specify)***		

**B19. Has the household suffered a fall in income due to any of the following household specific problems in the last 12 months?** (*Mark "YES" or "NO" for all options*)	**YES**	**NO**	
1. Loss of employment of any member…	1	2	**If any "YES" → B20**
2. Bankruptcy of a family business ……	1	2	
3. Illness or serious accident of a working member of the household	1	2	
4. Death of a working member of the household	1	2	
5. Abandonment by the household head	1	2	
6. Fire in the house/business/property	1	2	
7. Criminal act by household member	1	2	**Otherwise →B21**
8. Land dispute	1	2	
9. Loss of cash support or in-kind assistance	1	2	
10. Fall in prices of products of the household business.	1	2	
11. Loss of harvest..................................	1	2	
12. Loss of livestock....................................	1	2	
96. Other ……………………………	1	2	
**Other** (*Specify*)			

B20. How was it possible for the household to overcome this hardship? *(Multiple answers are allowed)*		Skip to question
A. Financial assistance from government agencies	**A**	
B. Financial assistance from NGOs/ religious organisations/ local community organisations	**B**	
C. Financial assistance from relatives / friends………….	**C**	
D. Took children out of school as could not afford it..	**D**	
E. Placed child(ren) in other household(s)	**E**	
F. Additional work hours by household members.	**F**	
G. Sold property/used savings…………	**G**	
H. Reduced household expenditures…….	**H**	
I. No serious impact ………	**I**	
J. Work of child	**J**	
K. Not solve until now	**K**	
U. Other ………………		
**Other** *(Specify)*		
**B21. Did any of your household members have any outstanding loans or obtain a new loan during the past 12 months?**		
1. Yes……………………..		**→B22**
2. No………………..…….		**→B28**

B22. What was the main reason for obtaining a loan?		
1. To meet essential household expenditures (buying food, child education etc).	**1**	
2. To buy vehicle (bike, motorbike, car) for household member	**2**	
3. To purchase/remodel/repair/construct a house.	**3**	
4. To meet health related expenditures for household members (medicine, doctor or hospital fees)	**4**	
5. To meet the following ritual expenditures: birth, funeral, and wedding	**5**	
6. To open/increase business	**6**	
7. To pay previous loan….	**7**	
96. Other……..	**96**	
**Other** *(Specify)*		
B23. Where did the household obtain the loan from? *(Multiple answers are allowed)*		
A. Government.........	**A**	
B. Bank/credit card…………………	**B**	
C. Micro-credit/finance groups.	**C**	
D. Employer/landowner……..	**D**	
E. Supplier of merchandise, equipment or raw materials	**E**	
F. A friend/relative of employer/landowner...	**F**	
G. Individual money lender……..	**G**	
H. A friend/relative of borrower	**H**	
U. Other………….		
**Other** *(Specify)*		

24. Was the debt paid back?		Skip to question
		→B25(A)
1. Yes, wholly	1	
2. Yes, partly	2	→B25(b)
3. No	3	

B25. A) How was the debt paid back? (*Read all the options and circle all the appropriate ones*)		
A. Cash, by borrowing money from someone else…	A	
B. Cash, by selling some assets..	B	
C. Cash, by getting income from work…	C	B26 (A)
D. Provide direct labour to the creditor by adult household member	D	B26 (A)
E. Provide direct labour to the creditor by child household member…	E	B26 (A)
F. In kind………………	F	B26 (A)
U. Other.................	U	
X. Don`t know....	X	
**B) How will the debt be paid back?** (*Read all the options and circle all the appropriate ones*)		
G. Cash, by borrowing money from someone else…	G	
H. Cash, by selling some assets..	H	B26 (B)
I. Cash, by getting income from work…	I	B26 (B)
J. Provide direct labour to the creditor by adult household member	J	B26 (B)
K. Provide direct labour to the creditor by child household member…	K	B26 (B)
L. In kind………………	L	
U. Other.................	U	
X. Don't know....	X	

**B26. A) Was any child withdrawn from school?**		
1. Yes……………………………………………	1	→B27
3 . No need to withdraw………..	3	→B28
**B) Will any child be withdrawn from school to pay the debt back?**		
1 Yes……………………………………………	1	
2. Maybe..	2	
3. No need to withdraw………..	**3**	→B28
**B27. Will the child/children withdrawn from school be sent back to school after the debt situation improves?**		
1. Yes	1	
2. Maybe	2	
3. No….	3	
**B28. What is the household's average monthly expenditure?** *(in local currency)* (*This question is to be recorded as expenditure incurred at the household level.*)	|___|___|___|___|	
**B.29. What are the household's sources of income?** *(Multiple answers are allowed)*		
A. Employment...	A	
B. Social transfers	B	
C. Rent/property	C	
D. Private transfers	D	
E. Remittances from any of H.H member living abroad	E	
F. Retirement **passion**	F	
U. Other	U	
**Other** *(Specify)*		

**B30. What is the household's average monthly income?**<br>*(in local currency)*	|___|___|___|___|	
**B31. Does the household receive any of the following :**	Yes No	
1. Pensions of the Ministry of Social Solidarity.	1 2	
2. Pensions Proclaimed by the Child Law	1 2	
3. Scholarships	1 2	
4. Pensions for Families of Civilians who died in military actions	1 2	
5. Support from Ministry of Social solidarity	1 2	
6. Pensions to families of recruited soldiers	1 2	
7. Support from the General Organization for Social solidarity	1 2	
8. Support from the Nasser Social Bank.	1 2	
9. Scholarship to the children of the families under the umbrella of Ministry of Social Solidarity (for basic and secondary education)	1 2	
10. Scholarships for visually impaired students in higher education	1 2	
11. Other	1 2	

Go to the 3rd part of the Questionnaire to interview each child (5-17)

SECTION IX — Educational Attainment of All Children (5-17) — 17 JUL 2007

<table>
<tr><th>Serial No in A1 →</th><th>|___|___|</th><th>|___|___|</th><th>|___|___|</th><th>|___|___|</th><th>|___|___|</th><th>|___|___|</th><th colspan="2">Skip to Question</th></tr>
<tr><td>Name of household member →</td><td></td><td></td><td></td><td></td><td></td><td></td><td colspan="2"></td></tr>
<tr><td>Age of household member →</td><td>|___|___|</td><td>|___|___|</td><td>|___|___|</td><td>|___|___|</td><td>|___|___|</td><td>|___|___|</td><td>Children
Aged 5-9 years</td><td>Children
Aged 10-17 years</td></tr>
<tr><td>C1. Can you read and write a short, simple statement with understanding in any language?
1. Yes……………………..
2. No…………………………..</td><td>1
2</td><td>1
2</td><td>1
2</td><td>1
2</td><td>1
2</td><td>1
2</td><td colspan="2"></td></tr>
<tr><td>C2. Are you attending school or pre-school during the current school year?
1. Yes…………………………….
2. No……………………………</td><td>1
2</td><td>1
2</td><td>1
2</td><td>1
2</td><td>1
2</td><td>1
2</td><td colspan="2">→C3
→C8</td></tr>
</table>

<table>
<tr><th>C3. What is the level of school and grade that you are currently attending? Level: (L) Grade (G)</th><th>L</th><th>G</th><th>L</th><th>G</th><th>L</th><th>G</th><th>L</th><th>G</th><th>L</th><th>G</th><th>L</th><th>G</th><th></th></tr>
<tr><td>1. Pre-school…………………</td><td>1</td><td>|___|</td><td>1</td><td>|___|</td><td>1</td><td>|___|</td><td>1</td><td>|___|</td><td>1</td><td>|___|</td><td>1</td><td>|___|</td><td></td></tr>
<tr><td>2. Primary……………………</td><td>2</td><td>|___|</td><td>2</td><td>|___|</td><td>2</td><td>|___|</td><td>2</td><td>|___|</td><td>2</td><td>|___|</td><td>2</td><td>|___|</td><td></td></tr>
<tr><td>3. preparatory…………………</td><td>3</td><td>|___|</td><td>3</td><td>|___|</td><td>3</td><td>|___|</td><td>3</td><td>|___|</td><td>3</td><td>|___|</td><td>3</td><td>|___|</td><td>C4</td></tr>
<tr><td>4. Secondary- general……….</td><td>4</td><td>|___|</td><td>4</td><td>|___|</td><td>4</td><td>|___|</td><td>4</td><td>|___|</td><td>4</td><td>|___|</td><td>4</td><td>|___|</td><td></td></tr>
<tr><td>5. Secondary-Technical………</td><td>5</td><td></td><td>5</td><td></td><td>5</td><td></td><td>5</td><td></td><td>5</td><td></td><td>5</td><td></td><td>}</td></tr>
<tr><td>6. Above intermediate…………</td><td>6</td><td></td><td>6</td><td></td><td>6</td><td></td><td>6</td><td></td><td>6</td><td></td><td>6</td><td></td><td></td></tr>
<tr><td>7. University or higher………</td><td>7</td><td></td><td>7</td><td></td><td>7</td><td></td><td>7</td><td></td><td>7</td><td></td><td>7</td><td></td><td></td></tr>
<tr><td>8. Non standard curriculum…</td><td>8</td><td>|___|</td><td>8</td><td>|___|</td><td>8</td><td>|___|</td><td>8</td><td>|___|</td><td>8</td><td>|___|</td><td>8</td><td>|___|</td><td>→C10</td></tr>
</table>

**C4. At what age did you begin primary school?** **(***If C3=1 write 95***)**<br><br>*(Age in completed years..)…….*	|___|___|	|___|___|	|___|___|	|___|___|	|___|___|	|___|___|		
**C5. Did you miss any school day during the past week?**<br>1. Yes……………..<br>2. No………………..	1<br>2	1<br>2	1<br>2	1<br>2	1<br>2	1<br>2	→C6<br>→C17	→C6<br>→C14
C6. How many school days did you miss during the past week?<br>***(write the number of days)……………………***	|___|	|___|	|___|	|___|	|___|	|___|		
**C7. Why did you miss school day(s) during the past week ?**<br>*(Read each of the following options and circle two most appropriate option)*<br>A. School vacation period……<br>B. Teacher was absent …………<br>C. Physical or emotional violence from teacher or peers<br>D. Bad weather conditions…….<br>E. To help family business…….<br>F. To help at home with household tasks ...<br>G. Working outside family business….<br>H. Illness/ Injury/disablement ………<br>U. Other ………………….	A<br>B<br>C<br>D<br>E<br>F<br>G<br>H<br>U	A<br>B<br>C<br>D<br>E<br>F<br>G<br>H<br>U	A<br>B<br>C<br>D<br>E<br>F<br>G<br>H<br>U	A<br>B<br>C<br>D<br>E<br>F<br>G<br>H<br>U	A<br>B<br>C<br>D<br>E<br>F<br>G<br>H<br>U	A<br>B<br>C<br>D<br>E<br>F<br>G<br>H<br>U	} C17	} C14
**Other** (Specify)……………………								

Serial No in A1 →	|__|__|	|__|__|	|__|__|	|__|__|	|__|__|	|__|__|	Skip to Question	
Name of household member →								
Age of household member →	|__|__|	|__|__|	|__|__|	|__|__|	|__|__|	|__|__|	Children Aged 5-9 years	Children Aged 10-17 years
**C8. Have you ever attended school?**								
1. Yes………………………..	1	1	1	1	1	1	→C10	
2. No……………………….	2	2	2	2	2	2	→C9	
C9. Why have you never attended school? (***read each of the following options and circle the most appropriate option)***								
1. Too young …………………..	01	01	01	01	01	01		
2. Disabled/ illness………………	02	02	02	02	02	02	C17	C14
3. Physical or emotional violence from teachers or peers	03	03	03	03	03	03		
4. I can't go to school at the same days of work	04	04	04	04	04	04		
5. No school/school too far…………………	05	05	05	05	05	05		
6. Cannot afford schooling…………	06	06	06	06	06	06		
7. Family can't afford other education costs	07	07	07	07	07	07		
8. Family did not allow schooling…	08	08	08	08	08	08		
9. Not interested in school…….	09	09	09	09	09	09		
10. Education not considered valuable.	10	10	10	10	10	10		
11. School not safe…………..	11	11	11	11	11	11		
12. To learn a job…………………	12	12	12	12	12	12		
13. To work for pay ……………	13	13	13	13	13	13		
14. My family not allow me to go to school because I'm a girl and my body grow up	14	14	14	14	14	14		
15. To work as unpaid worker in family business/farm ……….	15	15	15	15	15	15		

16. Help at home with household tasks…	16	16	16	16	16	16
17. I don't have a birth certificate	17	17	17	17	17	17
96. Other…………………………..	96	96	96	96	96	96
**Other**(Specify)						

**C10. What is the highest level of school and grade you have attended and completed successfully?** *Level (L) Grade (G)*	L	G	L	G	L	G	L	G	L	G	L	G
1. Pre-school…………………	1	|___|	1	|___|	1	|___|	1	|___|	1	|___|	1	|___|
2. Primary…………………….	2	|___|	2	|___|	2	|___|	2	|___|	2	|___|	2	|___|
3. Preparatory…………………	3	|___|	3	|___|	3	|___|	3	|___|	3	|___|	3	|___|
4. Secondary- general………..	4	|___|	4	|___|	4	|___|	4	|___|	4	|___|	4	|___|
5. Secondary-Technical………	5	|___|	5	|___|	5	|___|	5	|___|	5	|___|	5	|___|
6. Above intermediate………..	6	|___|	6	|___|	6	|___|	6	|___|	6	|___|	6	|___|
7. University or higher………	7	|___|	7	|___|	7	|___|	7	|___|	7	|___|	7	|___|
**8.** Non standard curriculum**…**	8	|___	8	|___	8	|___	8	|___	8	|___	8	|___

**C11. At what age did you begin primary school?** *(If C10=1 write 95) (Age in completed years)…………..*	|___|___|	|___|___|	|___|___|	|___|___|	|___|___|	|___|___|
**C12. At what age did you leave school?** *(Age in completed years)…………*	|___|___|	|___|___|	|___|___|	|___|___|	|___|___|	|___|___|

Serial No in A1 →	|__|__|	|__|__|	|__|__|	|__|__|	|__|__|	|__|__|	Skip to Question	
Name of household member →								
Age of household member →	|__|__|	|__|__|	|__|__|	|__|__|	|__|__|	|__|__|	Children Aged 5-9 years	Children Aged 10-17 years
**C13. Why did you leave school?** (*circle the most appropriate option*)								
1. Completed his/her compulsory schooling (if C10=X)	01	01	01	01	01	01		
2. Too old for school……….	02	02	02	02	02	02		
3. Disabled/ illness…………	03	03	03	03	03	03		
4. No school/school too far……………	04	04	04	04	04	04		
5. Cannot afford schooling…	05	05	05	05	05	05		
6. Family did not allow schooling.	06	06	06	06	06	06		
7. Poor in studies/not interested in school.	07	07	07	07	07	07		
8. Education not considered valuable	08	08	08	08	08	08		
9. School not safe………………	09	09	09	09	09	09	}(1-9) C17	
10. To learn a job………………	10	10	10	10	10	10		
11. To work for pay as employee or (as paid/ unpaid worker) in family business or farm..	11	11	11	11	11	11		
12. Help at home with household tasks.......................	12	12	12	12	12	12		
96. Other (Specify)…………………	96	96	96	96	96	96		
**Other**(Specify)								

**C14. Have you ever attended/are you currently attending a vocational / skills training course outside of school?**<br>1. Yes......................................<br>2. No………………………..	1<br>2	1<br>2	1<br>2	1<br>2	1<br>2	1<br>2		**→C15**<br>**→C17**
**C15. Have you /will you obtain a certificate for this vocational training?**<br>1. Yes ....<br>2. No………………………..	1<br>2	1<br>2	1<br>2	1<br>2	1<br>2	1<br>2		**→C16**<br>**→C17**
**C16. Describe subject of vocational training received/being received.**<br>(e.g Carpentry, Car repair, Nursing, etc)<br>*(If more than one then indicate the most important)*								
**For official use (OCCUPATION CODE)**	|__|__|__|__|	|__|__|__|__|	|__|__|__|__|	|__|__|__|__|	|__|__|__|__|	|__|__|__|__|		

SECTION X	Current Economic Activities Status of All Children (5-17)							
Serial No in A1 →	|__|__|	|__|__|	|__|__|	|__|__|	|__|__|	|__|__|	Skip to Question	
Name of household member →								
Age of household member →	|__|__|	|__|__|	|__|__|	|__|__|	|__|__|	|__|__|	Children Aged 5-9 years	Children Aged 10-17 years
Economic Activity								
C 17. Did you engage in any work at least one hour during the past week?<br>*(As employee, self employed, employer or unpaid family worker)*<br>1. Yes…………………<br>2. No………………………	1<br>2	1<br>2	1<br>2	1<br>2	1<br>2	1<br>2	→ C20<br>→ C18	
**C18. During the past week, did you do any of the following activities, even for only one hour?**<br>(*Read each of the following questions until the first affirmative response is obtained*)	**1= YES<br>2=NO**	**1= YES<br>2=NO**	**1= YES<br>2=NO**	**1= YES<br>2=NO**	**1= YES<br>2=NO**	**1= YES<br>2=NO**		
(a) **Run or do any kind of business, big or small, for himself/herself or with one or more partners?**<br>*Examples: Selling things, making things for sale, repairing things, guarding car, hairdressing, crèche business, taxi or other transport business, having a legal or medical practice, performing in public, having a public phone shop, barber, shoe shining , production of ghee, butter, cheese etc.*	|__|	|__|	|__|	|__|	|__|	|__|		
(b) **Do any work for a wage, salary, commission or any payment in kind (excl. domestic work)?**<br>*Examples: a regular job, contract, casual or piece work for pay, work in exchange for food or housing working in quarries, tanneries.*	|__|	|__|	|__|	|__|	|__|	|__|		

(c) Do any work as a domestic worker for a wage, salary or any payment in kind?	|___|	|___|	|___|	|___|	|___|	|___|	
(d) Help unpaid in a household business of any kind? *(Don't count normal housework.)* *Examples: Help to sell things, make things for sale or exchange, doing the accounts, cleaning up for the business, etc.*	|___|	|___|	|___|	|___|	|___|	|___|	If any "YES" → C20
(e) Do any work on his/her own or the household's plot, farm, food garden, or help in growing farm produce or in looking after animals for the household? *Examples: ploughing, harvesting, looking after livestock.*	|___|	|___|	|___|	|___|	|___|	|___|	
(f) Do any construction or major repair work on his/her own home, plot, or business or those of the household?	|___|	|___|	|___|	|___|	|___|	|___|	Otherwise
(g) Catch any fish, prawns, shells, wild animals or other food for sale or household food?	|___|	|___|	|___|	|___|	|___|	|___|	→C19
(h) Fetch water or collect firewood for household use?	|___|	|___|	|___|	|___|	|___|	|___|	
(i) Produce any other good for this household use? *Examples: clothing, furniture, clay pots, etc.*	|___|	|___|	|___|	|___|	|___|	|___|	

Serial No in A1 →	|___|___|	|___|___|	|___|___|	|___|___|	|___|___|	|___|___|	Skip to Question	
Name of household member →								
Age of household member →	|___|___|	|___|___|	|___|___|	|___|___|	|___|___|	|___|___|	Children Aged 5-9 years	Children Aged 10-17 years

**C19. Even though you did not do any of these activities in the past week, do you have a job, business, or other economic or farming activity that you will definitely return to?**<br>*(For agricultural activities, the off season in agriculture is not a temporary absence).*							
1. Yes…………………………..	1	1	1	1	1	1	**→C20**
2. No………………………..	2	2	2	2	2	2	**→C31**
**C20. Describe the main job/task you were performing e.g. carrying bricks; mixing baking flour; harvesting maize; etc.**<br>*("Main" refers to the work on which (NAME) spent most of the time during the week.)*							
**Job/Task**							
**OCCUPATION CODE**<br>**For official use**	|___|___|___|	|___|___|___|	|___|___|___|	|___|___|___|	|___|___|___|	|___|___|___|	
**C21. Describe briefly the main activity i.e. goods produced and services rendered where you are doing this job or task**							**Chek c17,c18,c19**<br>**If c17=2 and c18=2 in all opthions and c19=1**<br>**Then skip to c25**
**Activity / Type**							
**INDUSTRY CODE**<br>**For official use**	|__|__|__|__|	|__|__|__|__|	|__|__|__|__|	|__|__|__|__|	|__|__|__|__|	|__|__|__|__|	**→C33**

Serial No in A1 →	|__|__|		|__|__|		|__|__|		|__|__|		|__|__|		|__|__|		Skip to Question
Name of household member →													
Age of household member →	|__|__|		|__|__|		|__|__|		|__|__|		|__|__|		|__|__|		Children<br>Aged 10-17 years
**C22. In addition to your main work, did you do any other work during the past week?**													
1. Yes……………………………	1		1		1		1		1		1		
2. No…………………………….	2		2		2		2		2		2		
**C23. For each day worked during the past week how many hours did you actually work?**<br>*Main (M) Other (O)*	**M**	**O**	**M**	**O**	**M**	**O**	**M**	**O**	**M**	**O**	**M**	**O**	
1. Monday………………..	|__|__|	|__|	|__|__|	|__|	|__|__|	|__|	|__|__|	|__|	|__|__|	|__|	|__|__|	|__|	
2. Tuesday……………………….	|__|__|	|__|	|__|__|	|__|	|__|__|	|__|	|__|__|	|__|	|__|__|	|__|	|__|__|	|__|	
3. Wednesday………………….	|__|__|	|__|	|__|__|	|__|	|__|__|	|__|	|__|__|	|__|	|__|__|	|__|	|__|__|	|__|	
4. Thursday…………………	|__|__|	|__|	|__|__|	|__|	|__|__|	|__|	|__|__|	|__|	|__|__|	|__|	|__|__|	|__|	
5. Friday……………………….	|__|__|	|__|	|__|__|	|__|	|__|__|	|__|	|__|__|	|__|	|__|__|	|__|	|__|__|	|__|	
6. Saturday………………….	|__|__|	|__|	|__|__|	|__|	|__|__|	|__|	|__|__|	|__|	|__|__|	|__|	|__|__|	|__|	
7. Sunday……………………….	|__|__|	|__|	|__|__|	|__|	|__|__|	|__|	|__|__|	|__|	|__|__|	|__|	|__|__|	|__|	
**TOTAL**	|__|__|		|__|__|		|__|__|		|__|__|		|__|__|		|__|__|		

C24. During the past week when did you usually carry out these activities?							
For ALL children (including children attending school):							
A. During the day (between 6 a.m. and 6 p.m) …..	A	A	A	A	A	A	
B. In the evening or at night (after 6 p.m.)	B	B	B	B	B	B	
C. During both the day and the evening (for the entire day).	C	C	C	C	C	C	
D. On the week-end……………………..	D	D	D	D	D	D	
E. Sometimes during the day, sometimes in the evening	E	E	E	E	E	E	
ADDITIONAL: ***For children attending school ONLY (If C2=YES):***							
F. After school…………………	F	F	F	F	F	F	
G. Before school…………………..	G	G	G	G	G	G	
H. Both before or after school……….	H	H	H	H	H	H	
I. On the week-end…………………..	I	I	I	I	I	I	
J. During missed school hours/days…..	J	J	J	J	J	J	
K. I can't go to school at the same days of work	K	K	K	K	K	K	

Serial No in A1 →	|__|__|	|__|__|	|__|__|	|__|__|	|__|__|	|__|__|	**Skip to Question**
**Name of household member** →							
**Age of household member** →	|__|__|	|__|__|	|__|__|	|__|__|	|__|__|	|__|__|	**Children**<br>**Aged 10-17 years**

**C25. Where did you carry out your main work during the past week?**							
1. At (his/her) family dwelling…	01	01	01	01	01	01	
2. At dwelling for others	02	02	02	02	02	02	
3. Client's place ……………….	03	03	03	03	03	03	
4. Formal office …………….	04	04	04	04	04	04	
5. Factory / Atelier ………..	05	05	05	05	05	05	
6. Plantations / farm / garden…………….	06	06	06	06	06	06	
7. Construction sites…………….	07	07	07	07	07	07	
8. Mine / quarry………….	08	08	08	08	08	08	
9. Shop / kiosk / coffee house / restaurant / hotel	09	09	09	09	09	09	
10. Different places (mobile)………….	10	10	10	10	10	10	
11. Fixed market stall	11	11	11	11	11	11	
12. in street	12	12	12	12	12	12	
13. Pond / lake / river……………………	13	13	13	13	13	13	
96. Other…………………………….	96	96	96	96	96	96	
**Other** (specify)							
**C26. For your main job/work were you a/an….?**							
1. Employee………….	1	1	1	1	1	1	→C27
2. Own account worker (His/her own business without employees)	2	2	2	2	2	2	} **C28**
3. Employer (His/her own business with employees)	3	3	3	3	3	3	
4. Unpaid family worker….	4	4	4	4	4	4	**C30**
**C27. What was the mode of payment for the last payment period?**							
1. Piece rate………………………	01	01	01	01	01	01	
2. Hourly…………………………	02	02	02	02	02	02	
3. Daily……………………………	03	03	03	03	03	03	
4. Weekly…………………………	04	04	04	04	04	04	

5. Monthly..............................	05	05	05	05	05	05	
6. Upon completion of task.....	06	06	06	06	06	06	
96 Other (specify)....................	96	96	96	96	96	96	
**Other** (specify)							

**Serial No in A1** →	|___|___|	|___|___|	|___|___|	|___|___|	|___|___|	|___|___|	**Skip to Question**	
**Name of household member** →								
**Age of household member** →	|___|___|	|___|___|	|___|___|	|___|___|	|___|___|	|___|___|	**Children Aged 5-9 years**	**Children Aged 10-17 years**
**C28. What is your average monthly income from the main work?(*in local currency*)**	|___|___|___|	|___|___|___|	|___|___|___|	|___|___|___|	|___|___|___|	|___|___|___|		
**C29. What do you usually do with your earnings?** *(Multiple answers are allowed)*								
A. Give all/part of money to my parents/guardians…	A	A	A	A	A	A		
B. Employer gives all/part of money to my parents/guardians…	B	B	B	B	B	B		
C. Pay my school fees………	C	C	C	C	C	C		
D. Buy things for school …..	D	D	D	D	D	D		
E. Buy things for household	E	E	E	E	E	E		
F. Buy things for myself	F	F	F	F	F	F		
G. Save	G	G	G	G	G	G		
U. Other	U	U	U	U	U	U		
**Other (**specify**)**								

**C30. Why do you work?** *(Multiple answers are allowed)*								
A. Supplement family income…	A	A	A	A	A	A		
B. Help pay family debt…….	B	B	B	B	B	B		
C. Help in household enterprise…	C	C	C	C	C	C		
D. Learn skills…………	D	D	D	D	D	D		
E. Schooling not useful for future……..	E	E	E	E	E	E		
F. School too far / no school …………….	F	F	F	F	F	F		C33
G. Cannot afford school fees……	G	G	G	G	G	G		
H. Not interested in school……	H	H	H	H	H	H		
I. To temporarily replace someone unable to work.	I	I	I	I	I	I		
U. Other	U	U	U	U	U	U		
**Other (**specify**)**								
A. Job Search								
**C31. Were you seeking work ing the last week?**								
1. Yes……………………………..	1	1	1	1	1	1		
2. No……………………………	2	2	2	2	2	2		
**C32. At any time during the past 12 months did you engage in any work?**								
1. Yes……………………………..	1	1	1	1	1	1	**→C33**	
2. No……………………………	2	2	2	2	2	2	**→C41**	

SECTION XI	Health and Safety Issues about working children (5-17)							
**Serial No in A1** →	|___|___|	|___|___|	|___|___|	|___|___|	|___|___|	|___|___|	**Skip to Question**	
**Name of household member** →								
**Age of household member** →	|___|___|	|___|___|	|___|___|	|___|___|	|___|___|	|___|___|	**Children Aged 5-9 years**	**Children Aged 10-17 years**
**C33. Did you have any of the following in the past 12 months because of your work?** (*Read each of the following options and mark "YES" or "NO" for all options*)	**1= YES 2=NO**	**1= YES 2=NO**	**1= YES 2=NO**	**1= YES 2=NO**	**1= YES 2=NO**	**1= YES 2=NO**		
01. Superficial injuries or open wounds	01|___|	01|___|	01|___|	01|___|	01|___|	01|___|		
02. Fractures……….	02|___|	02|___|	02|___|	02|___|	02|___|	02|___|		
03. Dislocations, sprains or stains…	03|___|	03|___|	03|___|	03|___|	03|___|	03|___|		
04. Burns, corrosions, scalds or frostbite	04|___|	04|___|	04|___|	04|___|	04|___|	04|___|		
05. Breathing problems.................	05|___|	05|___|	05|___|	05|___|	05|___|	05|___|		
06. Eye problems...........................	06|___|	06|___|	06|___|	06|___|	06|___|	06|___|		
07. Skin problems…	07|___|	07|___|	07|___|	07|___|	07|___|	07|___|		
08. Stomach problems / diarrhea …	08|___|	08|___|	08|___|	08|___|	08|___|	08|___|		
09. Fever……….	09|___|	09|___|	09|___|	09|___|	09|___|	09|___|	If all **"NO"** → **C36**	
10. Extreme fatigue..	10|___|	10|___|	10|___|	10|___|	10|___|	10|___|		
96. Other (specify)........................	96|___|	96|___|	96|___|	96|___|	96|___|	96|___|		
**Other** (specify)							**Otherwise → C34**	

**C34. Think about your most serious illness/injury, how did this/these affect your work/schooling?**<br>1. Not serious- did not stop work/schooling.<br>2. Stopped work or school for a short time<br>3. Stopped work or school completely.	1<br>2<br>3	1<br>2<br>3	1<br>2<br>3	1<br>2<br>3	1<br>2<br>3	1<br>2<br>3	
**C35. Think about your most serious illness/injury, what were you doing when this happened?**							
**Job/Task**							
**OCCUPATION CODE**<br>**For Official use**	|___|___|___|	|___|___|___|	|___|___|___|	|___|___|___|	|___|___|___|	|___|___|___|	

**Serial No in A1** →	|___|___|	|___|___|	|___|___|	|___|___|	|___|___|	|___|___|	**Skip to Question**	
**Name of household member** →								
**Age of household member** →	|___|___|	|___|___|	|___|___|	|___|___|	|___|___|	|___|___|	**Children**<br>**Aged 5-9 years**	**Children**<br>**Aged 10-17 years**
**C36. Do you carry heavy loads at work?**<br>1. Yes...<br>2. No...	1<br>2	1<br>2	1<br>2	1<br>2	1<br>2	1<br>2		
**C37. Do you operate any machinery/heavy equipment at work?**<br>1. Yes…………………………..<br>2. No……………………………	1<br>2	1<br>2	1<br>2	1<br>2	1<br>2	1<br>2	→ C38<br>→ C39	

**C38.What type of tools, equipment or machines do you use at work?**<br><br>(*Write down 2 mostly used*)	1..............<br><br>2..............	1..............<br><br>2..............	1..............<br><br>2..............	1..............<br><br>2..............	1..............<br><br>2..............	1..............<br><br>2..............	
**C39. Are you exposed to any of the following at work?**<br>(*Read each of the following options and mark "YES" or "NO" for all options*)	**1= YES**<br>**2=NO**	**1= YES**<br>**2=NO**	**1= YES**<br>**2=NO**	**1= YES**<br>**2=NO**	**1= YES**<br>**2=NO**	**1= YES**<br>**2=NO**	
01. Dust, fumes,	01|___|	01|___|	01|___|	01|___|	01|___|	01|___|	
02. Fire, gas, flames............	02|___|	02|___|	02|___|	02|___|	02|___|	02|___|	
03. Loud noise or vibration......	03|___|	03|___|	03|___|	03|___|	03|___|	03|___|	
04. Extreme cold or heat	04|___|	04|___|	04|___|	04|___|	04|___|	04|___|	
05. Dangerous tools (knives etc)......	05|___|	05|___|	05|___|	05|___|	05|___|	05|___|	
06. Work underground.................	06|___|	06|___|	06|___|	06|___|	06|___|	06|___|	
07. Work at heights.......................	07|___|	07|___|	07|___|	07|___|	07|___|	07|___|	
08. Work in water/lake/pond/river............	08|___|	08|___|	08|___|	08|___|	08|___|	08|___|	
09. Workplace too dark or confined	09|___|	09|___|	09|___|	09|___|	09|___|	09|___|	
10. Insufficient ventilation\Bad smell..............	10|___|	10|___|	10|___|	10|___|	10|___|	10|___|	
11. Chemicals (pesticides, glues, etc.)..	11|___|	11|___|	11|___|	11|___|	11|___|	11|___|	
12. Explosives..................................	12|___|	12|___|	12|___|	12|___|	12|___|	12|___|	
13. getting extremely tired	13|___|	13|___|	13|___|	13|___|	13|___|	13|___|	
14. Bending for a long time	14|___|	14|___|	14|___|	14|___|	14|___|	14|___|	
15. no toilet in work place	15|___|	15|___|	15|___|	15|___|	15|___|	15|___|	
96. Other things, processes or conditions bad for your health or safety (specify).........................	96|___|	96|___|	96|___|	96|___|	96|___|	96|___|	
**Other** (specify)							

**C40. Have you ever been subject to the following at work?** (*Read each of the following options and mark "YES" or "NO" for all options*)	**1= YES 2=NO**	**1= YES 2=NO**	**1= YES 2=NO**	**1= YES 2=NO**	**1= YES 2=NO**	**1= YES 2=NO**	
1. Constantly shouted at ……………….	1|___|	1|___|	1|___|	1|___|	1|___|	1|___|	
2. Repeatedly insulted……..	2|___|	2|___|	2|___|	2|___|	2|___|	2|___|	
3. Beaten /physically hurt…	3|___|	3|___|	3|___|	3|___|	3|___|	3|___|	
4. Sexually abused (touched or done things to you that you did not want)	4|___|	4|___|	4|___|	4|___|	4|___|	4|___|	
7. Other (Specify)………………	7|___|	7|___|	7|___|	7|___|	7|___|	7|___|	
Other (specify)							

SECTION XII	Household Tasks of Children (5-17)						
Serial No in A1 →	|___|___|	|___|___|	|___|___|	|___|___|	|___|___|	|___|___|	Skip to Question	
Name of household member →								
Age of household member →	|___|___|	|___|___|	|___|___|	|___|___|	|___|___|	|___|___|	Children Aged 5-9 years	Children Aged 10-17 years
**C41. During the past week did you do any of the tasks indicated below for this household?** (*Read each of the following options and mark "YES" or "NO" for all options*)	1= YES 2=NO	1= YES 2=NO	1= YES 2=NO	1= YES 2=NO	1= YES 2=NO	1= YES 2=NO	If any "**YES**" →**C42**<br>Otherwise→ C44	
1. Shopping for household....	1|___|	1|___|	1|___|	1|___|	1|___|	1|___|		
2. Repair any household equipments	2|___|	2|___|	2|___|	2|___|	2|___|	2|___|		
3. Cooking..	3|___|	3|___|	3|___|	3|___|	3|___|	3|___|		
4. Cleaning utensils/house.................	4|___|	4|___|	4|___|	4|___|	4|___|	4|___|		
5. Washing clothes..........................	5|___|	5|___|	5|___|	5|___|	5|___|	5|___|		
6. Caring for children/old/sick.........	6|___|	6|___|	6|___|	6|___|	6|___|	6|___|		
7. Other household tasks.....	7|___|	7|___|	7|___|	7|___|	7|___|	7|___|		
Other (Specify)								
**C42. During each day of the past week how many hours did you do such household tasks?** (Record for each day separately)								
1. Monday………	|__|__|	|__|__|	|__|__|	|__|__|	|__|__|	|__|__|		
2. Tuesday……….	|__|__|	|__|__|	|__|__|	|__|__|	|__|__|	|__|__|		
3. Wednesday……….	|__|__|	|__|__|	|__|__|	|__|__|	|__|__|	|__|__|		
4. Thursday……………………	|__|__|	|__|__|	|__|__|	|__|__|	|__|__|	|__|__|		
5. Friday………………………..	|__|__|	|__|__|	|__|__|	|__|__|	|__|__|	|__|__|		
6. Saturday…………………..	|__|__|	|__|__|	|__|__|	|__|__|	|__|__|	|__|__|		

7. Sunday………………………	|__|__|	|__|__|	|__|__|	|__|__|	|__|__|	|__|__|	
**TOTAL**	|__|__|	|__|__|	|__|__|	|__|__|	|__|__|	|__|__|	
C43. During the past week when did you usually carry out these activities?							
For ALL children (including children attending school):							
A. During the day (between 6 a.m. and 6 p.m) …..	A	A	A	A	A	A	
B. In the evening or at night (after 6 p.m.)	B	B	B	B	B	B	
C. During both the day and the evening (for the entire day).	C	C	C	C	C	C	
D. On the week-end……………………..	D	D	D	D	D	D	
E. Sometimes during the day, sometimes in the evening	E	E	E	E	E	E	
ADDITIONAL: For children attending school ONLY (If C2=YES):							
F. After school.....................	F	F	F	F	F	F	
G. Before school......................	G	G	G	G	G	G	
H. Both before or after school…….....	H	H	H	H	H	H	
I. On the week-end………………….	I	I	I	I	I	I	
J. During missed school hours/days.....	J	J	J	J	J	J	
C44. Has (NAME) been interviewed in the company of an adult or an older child? *1. Yes* *2. No*	1<br>2	1<br>2	1<br>2	1<br>2	1<br>2	1<br>2	} **END** for this HH member. Go to the next child in Section II.

END OF INTERVIEW
