# Supplementary material for: Work-related injuries among 5 – 17 years-old working children in Egypt: findings from a national child labor survey
Source: BMC Public Health. 2022 Jul 7;22:1303. doi: 10.1186/s12889-022-13689-6 (PMC9260979; doi:10.1186/s12889-022-13689-6)
Supplement: Supplementary file 2 — Additional file 2: Table S1. Framework for identification of child labor (as described in the NCLS official report). Table S2. NCLS questionnaire items which were used to identify the work-related injuries. Table S3. NCLS questionnaire items which were used to identify working children. Table S4. Questions in NCLS questionnaire which describe the workplace hazards. [file 12889_2022_13689_MOESM2_ESM.docx]

**Table S1. Framework for identification of child labor (as described in the NCLS official report)**


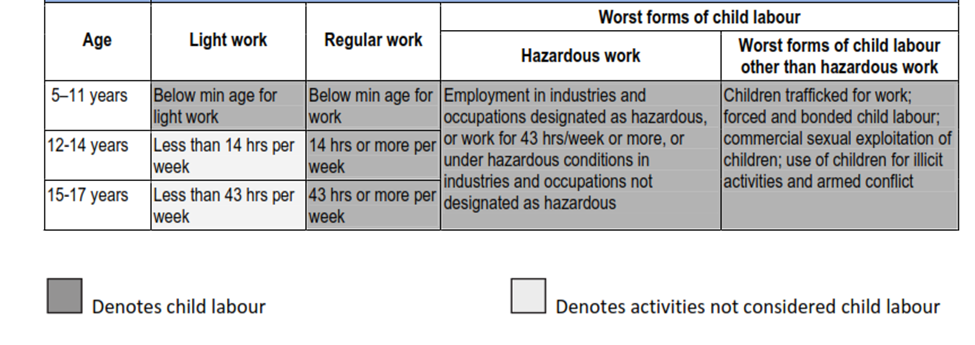


**Table S2. NCLS questionnaire items which were used to identify the work-related injuries**

| **C33. Did you have any of the following in the past 12 months because of your work?**  Read each of the following options and mark “YES” or “NO” for all options) |
| --- |
| 01. Superficial injuries or open wounds  02. Fractures ………  03. Dislocations, sprains or stains ….  04. Burns, corrosions, scalds or frostbite  06. Eye problems............................ |

**Table S3. NCLS questionnaire items which were used to identify working children**

| **Type of questionnaire** | **Question Number** | **Question Description** |
| --- | --- | --- |
| **Adult questionnaire** | **A18** | **Did (NAME) engage in any work at least one hour during the**  **past week?** (As employee, self-employed, employer or unpaid family worker) |
|  | **A19** | **During the past week did (NAME) do any of the following activities, even for only one hour? ….**  (Read each of the following questions until the first affirmative  response is obtained) |
|  | **A20** | **Even though (NAME) did not do any of these activities in the past week, does he/she have a job, business, or other economic or farming activity that he/she will definitely return to?** |
|  | **A24** | **During the past week, which of the following best describe (NAME) `s work situation at his/her main work?**  1. Employee  2. Own account worker (His/her own business without employees)  3. Employer (His/her own business with employees)  4. Unpaid family worker |
| **Child questionnaire** | **C 17** | **Did you engage in any work at least one hour during the past week?**  (As employee, self-employed, employer or unpaid family worker) |
|  | **C18** | **During the past week, did you do any of the following activities, even for only one hour?** …. |
|  | **C19** | **Even though you did not do any of these activities in the**  **past week, do you have a job, business, or other economic or**  **farming activity that you will definitely return to?**  (For agricultural activities, the off season in agriculture is not a  temporary absence). |
|  | **C22** | **In addition to your main work, did you do any other work during the past week?** |
|  | **C26** | **For your main job/work were you a/an….?**  1. Employee  2. Own account worker (His/her own business without employees)  3. Employer (His/her own business with employees)  4. Unpaid family worker |

**Table S4. Questions in NCLS questionnaire which describe the workplace hazards**

| **Type of Hazards** | **Question Number** | **Question Description** |
| --- | --- | --- |
| Ergonomic & Safety hazards | C36 | 'carry heavy loads at work’ |
|  | C37 | 'operate machinery/heavy equipment' |
|  | C39E | 'dangerous tools (knives, etc.) at work' |
|  | C39I | 'workplace too dark or confined' |
|  | C39M | 'exhaustion' |
|  | C39N | 'bending for long time' |
| Chemical hazards | C39A | 'dust, fumes at work' |
|  | C39B | 'fire, gas, flames at work' |
|  | C39K | 'chemicals (pesticides, glues, etc.)' |
|  | C39L | 'explosives' |
|  | C39J | 'insufficient ventilation' |
| Physical hazards | C39C | 'loud noise or vibration at work' |
|  | C39D | 'extreme cold or heat at work'; |
|  | C39F | 'working underground' |
|  | C39G | 'working at heights' |
|  | C39H | 'work in water/lake/pond/river' |
